# TCA-phospholipid-glycolysis targeted triple therapy effectively suppresses ATP production and tumor growth in glioblastoma

**DOI:** 10.7150/thno.74197

**Published:** 2022-10-03

**Authors:** Shixue Yang, Jixing Zhao, Xiaoteng Cui, Qi Zhan, Kaikai Yi, Qixue Wang, Menglin Xiao, Yanli Tan, Biao Hong, Chuan Fang, Chunsheng Kang

**Affiliations:** 1 Department of Neurosurgery, Tianjin Medical University General Hospital, Lab of Neuro-oncology, Tianjin Neurological Institute, Tianjin, 300052, China.; 2 Key Laboratory of Post-Neuro Injury Neuro-repair and Regeneration in Central Nervous System, Ministry of Education and Tianjin City, Tianjin, 300052, China.; 3 Tianjin Key Laboratory of Composite and Functional Materials, School of Material Science and Engineering, Tianjin University, Tianjin, 300072, China.; 4 Department of Neuro-Oncology and Neurosurgery, Tianjin Medical University Cancer Institute and Hospital, National Clinical Research Center for Cancer, Key Laboratory of Cancer Prevention and Therapy of Tianjin, Tianjin's Clinical Research Center for Cancer, Tianjin 300060, China.; 5 Department of Neurosurgery, Affiliated Hospital of Hebei University, Hebei Key Laboratory of Precise Diagnosis and Treatment of Glioma, Baoding, 071000, China.; 6 Department of Pathology, Affiliated Hospital of Hebei University, Department of Pathology, Hebei University School of Basic Medical Sciences, Baoding, 071000, China.

**Keywords:** glioblastoma, ATP production, energy metabolism, convection-enhanced delivery.

## Abstract

**Rationale:** Glioblastoma (GBM) displays a complex metabolic reprogramming in cancer cells. Adenosine triphosphate (ATP) is one of the central mediators of cell metabolism and signaling. GBM cells generate ATP by glycolysis and the tricarboxylic acid (TCA) cycle associated with oxidative phosphorylation (OXPHOS) through the breaking-down of pyruvate or fatty acids to meet the growing energy demand of cancer cells. Therefore, it's urgent to develop novel treatments targeting energy metabolism to hinder tumor cell proliferation in GBM.

**Methods:** Non-targeted metabolomic profiling analysis was utilized to evaluate cell metabolic reprogramming using a small molecule inhibitor (SMI) EPIC-0412 treatment. Cellular oxygen consumption rate (OCR) and the total proton efflux rate (PER), as well as ATP concentration, were tracked to study metabolic responses to specifically targeted inhibitors, including EPIC-0412, arachidonyl trifluoromethyl ketone (AACOCF3), and 2 deoxy-D-glucose (2-DG). Cancer cell proliferation was assessed by CCK-8 measurements and colony formation assay. Additionally, flow cytometry, immunoblotting (IB), and immunofluorescence (IF) analyses were performed with GBM cells to understand their tumorigenic properties under treatments. Finally, the anticancer effects of this combination therapy were evaluated in the GBM mouse model by convection-enhanced delivery (CED).

**Results:** We found that SMI EPIC-0412 could effectively perturb the TCA cycle, which participated in the combination therapy of cytosolic phospholipase A2 (cPLA2)-inhibitor AACOCF3, and hexokinase II (HK2)-inhibitor 2-DG to disrupt the GBM energy metabolism for targeted metabolic treatments. ATP production was significantly declined in glioma cells when treated with monotherapy (EPIC-0412 or AACOCF3), dual therapy (EPIC-0412 + AACOCF3), or triple therapy (EPIC-0412 + AACOCF3 +2-DG) regimen. Our experiments revealed that these therapies hindered glioma cell proliferation and growth, leading to the reduction in ATP production and G0/G1 cell cycle arrest. We demonstrated that the combination therapy effectively extended the survival of cerebral tumor-bearing mice.

**Conclusion:** Our findings indicate that the TCA-phospholipid-glycolysis metabolism axis can be blocked by specific inhibitors that significantly disrupt the tumor energy metabolism and suppress tumor proliferation *in vitro* and* in vivo*, suggesting that targeting ATP synthesis inhibition in cancer cells might be an attractive therapeutic avenue in GBM management.

## Introduction

Glioblastoma (GBM) is the most prevalent and aggressive type of malignant primary intracranial tumor. It's also one of the most refractory cancers in humans. The median survival time of patients with primary glioblastoma is less than 16 months after diagnosis, and indeed the five-year survival rate is around 10% 1, 2. The long-term survival of GBM patients urgently demands in-depth scientific investigations to better understand the underlying mechanisms that support the growth and malignancy of GBM tumors. Recent studies have found that aberrant energy metabolism orchestrates tumorigenesis, immune responses, and epigenetic regulations 3, 4.

Adenosine triphosphate (ATP) plays a central role in energy metabolism and cell signaling 5-7. In the last century, Otto Warburg observed that most of the ATP required for anabolic processes in tumor cells was created by aerobic glycolysis, known as the Warburg effect 8. Additionally, fatty acid oxidation (FAO) also contributes to the growth and proliferation of glioma cells 9. The tricarboxylic acid (TCA) cycle associates with oxidative phosphorylation (OXPHOS) to synthesize ATP molecules in mitochondria 10. GBM reprograms these energy metabolic pathways to produce a higher proportion of ATP supporting the rapid growth process of cancer cells 11. Phospholipid metabolism could be promoted by the elevated expression of polymerase 1 and transcript release factor (PTRF), which increases the free fatty acid (FFA) content to promote FAO in GBM 12, 13. GBM pathogenesis physiologically depends on specific energy pathways, which can be harnessed for therapeutic targeting. Mechanistically, therapeutics should be designed to limit ATP production to the greatest extent possible, particularly in GBM tumor cells. Furthermore, dual inhibition of glycolysis and FAO, reported in previous animal studies, has demonstrated that combination therapy could significantly block the major ATP production pathways in glioma cells 14-16. Hence, we hypothesized that the combination therapy targeting both glycolysis and FAO pathways might exert robust inhibitory effects on the proliferation of GBM cells, instead of monotherapy strategies.

Noncoding RNA, DNA methylation, and histone methylation and acetylation are amongst the crucial epigenetic mechanisms that play critical roles in the functional modifications promoting tumor cell metabolic reprogramming 17. Long noncoding RNAs (lncRNAs), involved in fundamental biochemical processes, are a heterogeneous group of non-protein-coding transcripts with lengths longer than 200 nucleotides 18-20. LncRNA modulates energy metabolism and cancer progression through the epigenetic regulation of key metabolism-associated genes 21, 22. Mechanistic studies of the lncRNA HOX transcript antisense RNA (HOTAIR) have revealed that the 5' region of HOTAIR mediates gene silencing via Enhancer of Zeste Homolog 2 (EZH2), a subunit of Polycomb Repressive Complex 2 (PRC2) protein, driven trimethylation of histone H3 at Lys27 (H3K27me3), while the 3' domain regulates gene activation through Lysine-Specific Demethylase 1 (LSD1) of LSD1/CoREST/REST complex-mediated demethylation of dimethylated histone H3 at Lys4 (H3K4me2) 23-25. It's worth mentioning that further understanding of energy metabolism and the influence of epigenetic inheritance will be highly helpful in developing novel therapies to combat this extremely invasive and heterogeneous disease.

Hence, we demonstrated that EPIC-0412 (EPIC) could hinder the GBM tumorigenesis by effectively impairing the interaction between lncRNA HOTAIR and EZH2, and further modulating the TCA cycle. To find a comprehensive approach to inhibit ATP production, a cPLA2 inhibitor arachidonyl trifluoromethyl ketone (AA, short for AACOCF3), and a HK2 inhibitor nonmetabolizable glucose analog 2 deoxy-D-glucose (2-DG) were used in this study. AA hinders the production of FFAs during phospholipid metabolism, while 2-DG partakes in the glycolysis blocking via selective inhibition of HK2. Here, we investigated the role of ATP production on cell cycle progression in GBM cell proliferation *in vitro*. Then we exploited patient-derived GBM cells, along with convection-enhanced delivery (CED) by micro-osmotic pumps implanted in the mouse brain tumor to explore the therapeutic outcomes of targeted reprogramming of energy metabolism pathways in GBM tumors. Finally, we demonstrated that a drastically high amount of ATP produced within mitochondria and through glycolysis in cancer cells could be significantly decreased by targeting the TCA-phospholipid-glycolysis axis in a triple therapy approach, leading to a robust inhibition of tumor growth and improved survival outcomes of tumor-bearing mice.

## Materials and Methods

### Cell culture, lentiviruses, and drugs

TBD0220, is a glioma cell line that derived from a female GBM patient who with no history of radiation therapy or chemotherapy and underwent surgery at Hebei University-affiliated hospital 26. After several generations of culture and passage, these cells tend to be stable and immortal and have been maintained in DMEM/F12 medium supplemented with 10% fetal bovine serum (FBS). Human GBM cell line U87MG (ATCC, USA) was cultured in DMEM with 10% FBS. Lentiviral particles were obtained from GENECHEM (Shanghai, China). Small interfering RNAs (siRNAs) targeting ATF3 were purchased from Gene Pharma (Suzhou, China). The sequences of siRNAs against ATF3 mRNA, are as follows: siRNA#1: 5'-GAGGCGACGAGAAAGAAAUTT-3'; siRNA#2: 5'-GCCGAAACAAGAAGAAGGATT-3'; and negative control (NC): 5'-UUCUCCGAACGUGUCACGUTT-3'. Glioma cells were treated with EPIC (WuXi AppTec, China) at a final concentration of 20 μM; AACOCF3 (Cayman Chemical, Cat# 62120) at a final concentration of 25 μM; and 2-DG (Sigma-Aldrich, Cat# D8375) at a final concentration of 50 mM. Dimethyl sulfoxide (DMSO; Fisher Scientific, Cat# 85190) was used as the solvent.

### The competing endogenous RNA (ceRNA) microarray assay

The human ceRNA microarray was performed as described elsewhere 27. The data analysis for RNA sequencing results from the total RNA from the glioblastoma cells (TBD0220 cell line) was carried out by OE Biotechnology Co., Ltd., (Shanghai, China).

### Untargeted metabolomic analysis

Extraction and sample preparations were performed following a published protocol by Shanghai Lu-Ming Biotech Co. Ltd. (Shanghai, China, 2021). Non-targeted metabolite analysis was performed by OE Biotech (Shanghai, China). A Dionex Ultimate 3000 RS UHPLC system fitted with a Q-Exactive quadrupole-Orbitrap mass spectrometer and equipped with heated electrospray ionization (ESI) source (Thermo Fisher Scientific, Waltham, MA, USA) was used to analyze the metabolic profiling in both ESI-positive and ESI-negative ion modes. An ACQUITY UPLC BEH C18 column (1.7 μm, 2.1 × 100 mm^2^) was employed in both positive and negative modes. All the samples were kept at 4℃ during the analysis. QCs were conducted at regular intervals (every 10 samples) during the analytical run to provide a reproducible dataset. The progenesis QI software (Waters Corporation, Milford, USA) was used for analyzing the acquired LC-MS raw datasets.

### Seahorse XFe24 extracellular flux analysis

Seahorse extracellular flux analyzer (Seahorse Bioscience) was used to measure cellular OCR and cellular PER as the manufacturer's protocol as reported 12, 28. Data were analyzed by the Agilent Seahorse XFe24 Wave software. The data of OCR and glycolytic rate measurements were standardized to the cell count. Both OCR and PER were expressed as pmol/min/cells.

### ATP and lactic acid concentration measurements

The ATP assay kit was provided by Beyotime (Shanghai, China). The ATP concentration measurement was carried out according to the manufacturer's protocol. NMR profiles of lactic acid metabolites were detected as follows: 400 µL of cell culture medium and 400 µL of buffer were thoroughly mixed, finally, 600 µL of the mixture was used to detect the concentration of diverse metabolites on a 600 MHz NMR Avance III HD spectrometer. Following Fourier processing, the FIDs were shown as spectra. TopSpin software was used for automatic phase and baseline corrections using the Bruker IVDr protocol. The concentration of metabolites was expressed as mmol/L.

### Chromatin immunoprecipitation (ChIP) and ChIP-qPCR assays

Millipore Magna ChIP™ A/G Chromatin Immunoprecipitation kit (Cat# 17-10085) was used for ChIP assay as per the manufacturer's instructions 29. The ChIP eluates were purified using a Qiagen PCR purification kit (Cat# 28104). Then precipitated DNA fragments were quantified by qRT-PCR analysis. Samples were analyzed using the following primers:

H3K27Me3/ATF3-1F: 5´-ATCCACGGGCAGTCAAGAAG-3´; H3K27Me3/ATF3-1R: 5´-CCGAGATTCGAGCTGAGACC-3´; H3K27Me3/ATF3-2F: 5´-ACTTCAAGTGAGACCCAGGC-3´; H3K27Me3/ATF3-2R: 5´-CTCCTTTCCACCTGCCTCTG-3´; RNA Pol II/ATF3-1F: 5´-GCGACAAGTATCCTCCCAGC-3´; RNA Pol II/ATF3-1R: 5´-TCTTTTCCCAAAGCCCTGTGT-3´; RNA Pol II/ATF3-2F: 5´-CCAGGCCTCCAAACAAAGAC-3´; RNA Pol II/ATF3-2R: 5´-AGGCCAATGAAATCTGCCCTT-3´; ATF3/SDHA-1F: 5´-GCTCACACCGGACACTTTCA-3´; ATF3/SDHA-1R: 5´-TGCATTCATCACCACCACGA-3´; ATF3/SDHA-2F: 5´-GCAGCATTTTGACAGGCAGA-3´; ATF3/SDHA-2R: 5´-CTGCATTCATCACCACCACG-3´.

### RNA extraction and quantitative RT-PCR

According to the manufacturer's instructions, total RNA was isolated from cells using TRIzol^TM^ Reagent (Invitrogen, Cat# 15596018). The 2^-ΔΔCt^ method was used to calculate relative gene expressions. The mean and standard deviation (SD) of three independent experiments were used to represent the data. Primers used include: ATF3-1F: 5´-CGCTGGAATCAGTCACTGTCAG-3´; ATF3-1R: 5´-CTTGTTTCGGCACTTTGCAGCTG-3´; ATF3-2F: 5´-CCTCTGCGCTGGAATCAGTC-3´; ATF3-2R: 5´-TTCTTTCTCGTCGCCTCTTTTT-3´; SDHA-1F: 5´-CAGCATGTGTTACCAAGCTGT-3´; SDHA-1R: 5´-GGTGTCGTAGAAATGCCACCT-3´; SDHA-2F: 5´-TGGCATTTCTACGACACCGTG-3´; SDHA-2R: 5´-GCCTGCTCCGTCATGTAGTG-3´; SDHB-F: 5´-ACAGCTCCCCGTATCAAGAAA-3´; SDHB-R: 5´-GCATGATCTTCGGAAGGTCAA-3´; SDHC-F: 5´-TAGGTTCAAACCGTCCTCTGT-3´; SDHC-R: 5´-GAGAGACCCCTGCACTCAAAG-3´; SDHD-F: 5´-TTGCTCTGCGATGGACTATTCC-3´; SDHD-R: 5´-CAAGGCATCCCCATGAACAT-3´; β-tubulin-F: 5´-TGGACTCTGTTCGCTCAGGT-3´; β-tubulin-R: 5´-TGCCTCCTTCCGTACCACAT-3´*.*

### Western blotting (WB) analysis

Protein extraction and WB analysis were carried out following the manufacturer's protocol. The primary antibodies used in this study included: anti-H3K27me3 (CST, Cat# 9733); anti-RNA polymerase II (Sigma-Aldrich, Cat# 05-623); anti-ATF3 (CST, Cat# 18665); anti-SDHA (Protein-tech, Cat# 14865-1-AP); anti-β-actin (CST, Cat# 3700); anti-β-tubulin (CST, Cat# 2146); anti-p21 Waf1/Cip1 (CST, Cat# 2947); anti-CDK4 (Affinity Biosciences, Cat# DF6102); anti-CDK6 (Abcam, Cat# ab124821); anti-cyclin D1 (Affinity Biosciences, Cat# AF0931); anti-Retinoblastoma (Rb) (Affinity Biosciences, Cat# DF6840); anti-Phospho-Retinoblastoma (p-Rb) (Affinity Biosciences, Cat# AF3103); anti-cyclin E1 (Affinity Biosciences, Cat# AF0144); anti-cyclin A (Affinity Biosciences, Cat# AF0142); anti-HK2 (CST, Cat# 2867). After probing with primary antibodies, corresponding horseradish peroxidase (HRP)-conjugated secondary antibodies were used to visualize the protein bands.

### Cell viability, colony formation assay, and cell cycle analysis

For the cell viability assay, Cell Counting Kit-8 (CCK-8; Dojindo; Japan) was used to evaluate the proliferation of GBM cells as per the manufacturer's protocol. The assay was repeated thrice. For the colony formation assay, GBM cells were cultured in a medium with various treatment conditions for around 14 days after being seeded onto 6-well plates in triplicates, with approximately 500 cells per well. Subsequently, cell colonies were fixed with a 4% paraformaldehyde (PFA) solution, then stained with crystal violet. The images of colony plaques were taken using a camera, and the number of colonies was analyzed by ImageJ. Cell Cycle Analysis Kit (Beyotime, China) was used to measure the cell cycle distribution pattern, following the manufacturer's protocol. After staining, cells were analyzed by flow cytometry.

### Confocal laser scanning microscope (CLSM)

Briefly, GBM cells were grown on coverslips, fixed with 4% PFA for 30 min and blocked with 10% FBS for 1 h. Then, cells were incubated with a primary antibody (anti-CDK6 and phalloidin) at 4°C overnight. Then, the target protein levels were detected by Alexa Fluor488- or 594- conjugated secondary antibody (Life Technologies, USA). Nuclei were counterstained with DAPI (Solarbio; Cat# C0060). The images were captured by Olympus FluoView 2000 confocal microscope (Olympus, Tokyo, Japan). The quantification of the CDK6 protein band was measured by ImageJ.

### Intracranial mouse tumor model and treatments

To establish the cerebral tumor mouse model, 2×10^5^ patient-derived GBM cells were implanted into the brains of four-week-old female BALB/C nude mice by the stereotactic method. When tumors were detected by IVIS imaging, mice were divided randomly (7 mice in each group) into three groups, namely EPIC, AA, and EPIC+AA groups. Then mice were treated every day by oral gavage for 2 weeks (EPIC 15 mg/kg; AA 25 mg/kg; EPIC 15 mg/kg + AA 25 mg/kg). The intracranial tumor formation was examined by parietal bioluminescence on days 7, 14, and 21. Kaplan-Meier survival method was applied to plot the animal's survival curves. All animal experiments were approved by the Animal Ethics and Welfare Committee (AEWC) of Hebei University.

### Hematoxylin and Eosin (H&E) staining and immunohistochemistry (IHC)

H&E staining and IHC assays were carried out as previously reported 30. The primary antibodies included anti-Ki67 (CST, Cat# 9449), and anti-CDK6 (Abcam, Cat# ab124821). The H&E and IHC images were captured by a brightfield microscope (CX41, Olympus).

### *In vivo* procedure for Alzet micro-osmotic pump implantation

The standard procedure of Alzet micro-osmotic pump implantation was followed as previously reported 30. Approximately 7 days after intracranial tumor cell implantation and brain tumor detection by IVIS imaging, mice were randomly divided into three groups. The Alzet micro-osmotic pump (ALZET, model 1004) contained different treatment solutions: 1) vehicle (DMSO); 2) EPIC (15 mg/kg = 68 μg/μL in total), or AA (25 mg/kg = 114 μg/μL in total); and 3) EPIC + AA + 2-DG (15 mg/kg = 68 μg/μL in total). Tumor growth was detected with bioluminescence imaging on days 7, 14, 21, and 28. The Kaplan-Meier survival curve was created and used to observe the animal's survival status. All animal experiments were approved by the AEWC of Hebei University.

### TCGA Dataset

The gene expression profiles of activating transcription factor 3 (ATF3) and succinate dehydrogenase complex flavoprotein subunit A (SDHA) in GBM datasets were downloaded from TCGA (https://www.cancer.gov/).

### Statistical analyses

GraphPad Prism 8.0 software was used to conduct statistical analyses. All data are presented as mean ± SD. The paired Student's t-test was used to compare experimental and control groups. For multiple experimental groups, one-way or two-way analysis of variance (ANOVA) was applied. Significance was defined as *p < 0.05, **p < 0.01, ***p < 0.001, ****p < 0.0001, and ns = nonsignificant.

## Results

### EPIC-0412 disrupts TCA cycle in glioma cells

**Figure 1:**
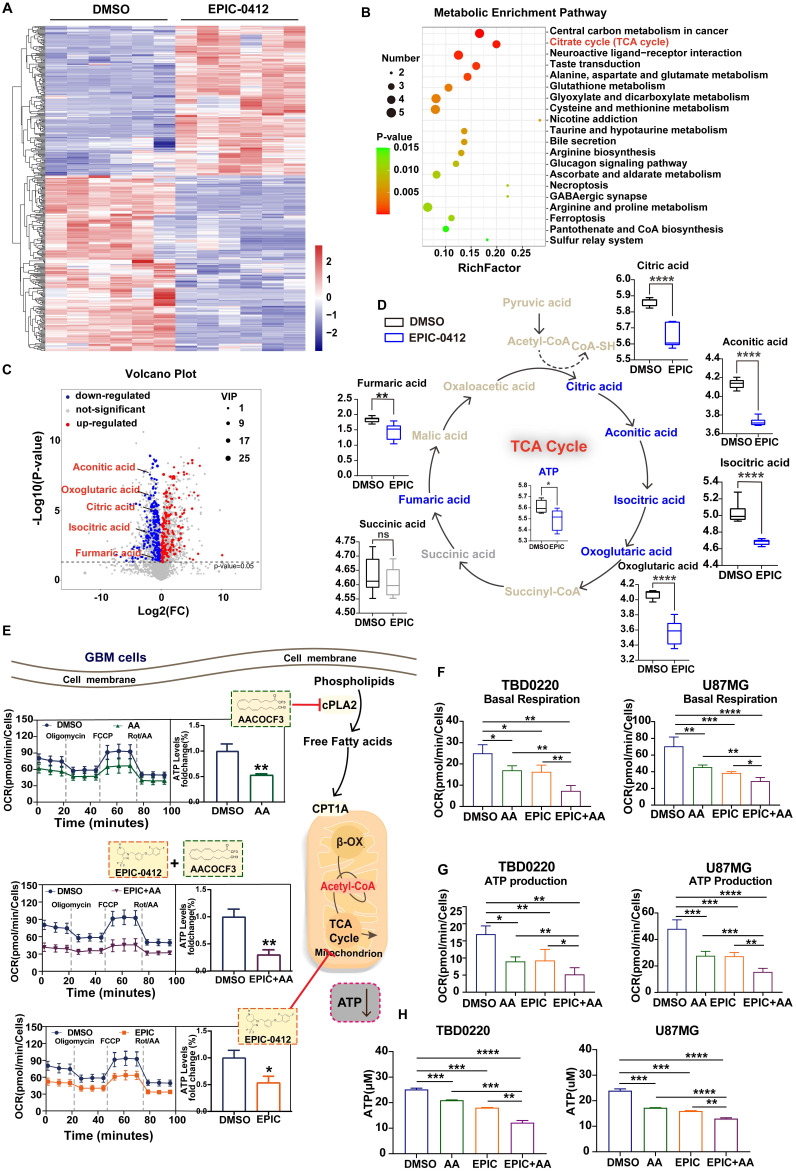
** EPIC and AA disrupt the TCA cycle as well as phospholipid metabolism and account for a synergistic effect on ATP production inhibition. (A)** Heatmap of metabolite changes in TBD0220 cells treated with or without EPIC (20 µM) detected via non-targeted metabolomic analysis (DMSO group, n = 6; EPIC group, n = 6). **(B)** Pathway analysis of significantly affected metabolites in TBD0220 cells treated with EPIC in (A) (FC > 2, p < 0.05, and VIP > 1). **(C)** A volcano plot analysis was performed on a non-targeted metabolomic study. Key metabolites, including citric acid, aconitic acid, isocratic acid, α-oxoglutaric acid, and fumaric acid, were significantly downregulated following EPIC treatment in GBM cells. Each spot represents a metabolite, upregulated metabolites are shown in red, and downregulated metabolites are shown in blue. Significant changes are denoted by FC > 2, p < 0.05, and VIP > 1. **(D)** Representation of the TCA cycle and energy metabolites, box plots show relative abundances of metabolites after normalization. Metabolites shown in blue were decreased, metabolites shown in gray were non-significant, and metabolites indicated in brown remained undetected. (n = 6 per group) **(E)** The general scheme of the TCA cycle and lipid metabolism as the therapeutic target of EPIC (20 µM) or/and AA (25 µM) for 24 h in TBD0220 cells. The oxygen consumption rate (OCR) of TBD0220 cells was measured on a Seahorse Flux Analyzer (n = 3-4). **(F-G)** Measurements of basal respiration and ATP production in TBD0220 (F) or U87MG (G) cells. **(H)** Measurement of the intracellular total ATP concentration in TBD0220 or U87MG cells (n = 3 per group). All data are shown as the mean values ± SD, *p* values are based on Student's *t-test*, one-way ANOVA. ****p < 0.0001, ***p < 0.001, **p < 0.01, *p < 0.05; ns, nonsignificant.

Cellular metabolism takes part in the regulation of the growth and proliferation of tumor cells by altering the tumor microenvironment and epigenetic regulations 31-33. The small-molecule inhibitor EPIC, is a derivative of AC1Q3QWB (AQB). Functionally, AQB, developed by Tianjin Neurological Institute, could disrupt the lncRNA HOTAIR-EZH2 interaction and have potent anti-tumor efficacies both *in vitro* and *in vivo* 34. Compared with the DMSO-treated cells, a distinct metabolite profile was observed in TBD0220 cells after treatment with EPIC for 48 h (**Figure 1A**). Pathway analysis of significantly altered metabolites identified carbon metabolism, TCA cycle metabolism, and neuroactive ligand-receptor interaction as the three most affected metabolic pathways **(Figure 1B)**, which eventually directed us to focus on the TCA cycle regulation because of its key roles in both catabolic and anabolic activities in cells. We found that TCA cycle-related metabolites, including citric acid, aconitic acid, isocratic acid, α-oxoglutaric acid, and fumaric acid, were significantly downregulated by EPIC treatment, as shown in the volcano plot (**Figure 1C**). Furthermore, we noted a potent reduction in ATP levels in GBM cells following the EPIC exposure (**Figure 1D**). To verify that these observations were not accidental, we performed extracellular flux analysis by measuring OCR in glioma cells. The workflow scheme of classical XF Mito Stress assay is described in **Figure S1A**. The OCR measurements of TBD0220 (**Figure S1B**), and U87MG (**Figure S1C**) cell lines confirmed that the mitochondrial ATP synthesis was significantly obstructed in EPIC-treated cells. Our results showed that the mitochondrial ATP levels in TBD0220 and U87MG cells were reduced by ~44% (**Figure S1D**) and ~46% (**Figure S1E**), respectively. Taken together, these results demonstrated that EPIC influenced the energy metabolism in GBM cells.

Additionally, we have shown that vital enzymes like cPLA2 could fuel endocytosis, ATP production, and cell proliferation in glioma cells through phospholipid reprogramming 12. Therefore, AA, a selective inhibitor of cPLA2, could inhibit ATP production and hinder the growth and proliferation of glioma cells. Due to its evident efficacy in the suppression of ATP production, AA was considered a powerful candidate to study the mechanism of tumor growth retardation in our model. Furthermore, OCRs were measured in TBD0220 cells following the monotherapy with AA or EPIC and dual therapy of EPIC and AA. We noted that the ATP production was differentially modulated in different treatment modes (**Figure 1E**). Besides, we observed that rates of basal respiration and ATP production were both decreased in TBD0220 and U87MG cells under EPIC or/and AA treatments contrasted with control cells **(Figure 1F-G)**. Notably, the combination therapy with EPIC and AA was more effective than monotherapy (EPIC or AA), showing about a 70% decrease in ATP production from mitochondrial respiration in tumor cells (**Figure S1D-E**). We found significantly decreased intracellular contents of total ATP in glioma cells when subjected to our therapies. Moreover, the dual therapy of EPIC and AA was more effective declining the ATP production than the monotherapy of either EPIC or AA, which was consistent with OCR measurements **(Figure 1H)**.

In summary, the mitochondrial TCA cycle was disrupted by EPIC, and the FAO was partially blocked by AA, leading to the overall slowdown of cellular metabolisms. AA-targeted phospholipid metabolism alteration might add a synergistic effect to EPIC-mediated ATP production inhibition. Moreover, our findings indicated that FFAs could be used as a fuel to mitigate energy demand-related issues in glioma cells. Altogether, these data suggest that EPIC and AA are promising anti-GBM agents when used in combination.

### H3K27me3-ATF3-SDHA axis accounts for deteriorated TCA cycle under EPIC treatment

**Figure 2:**
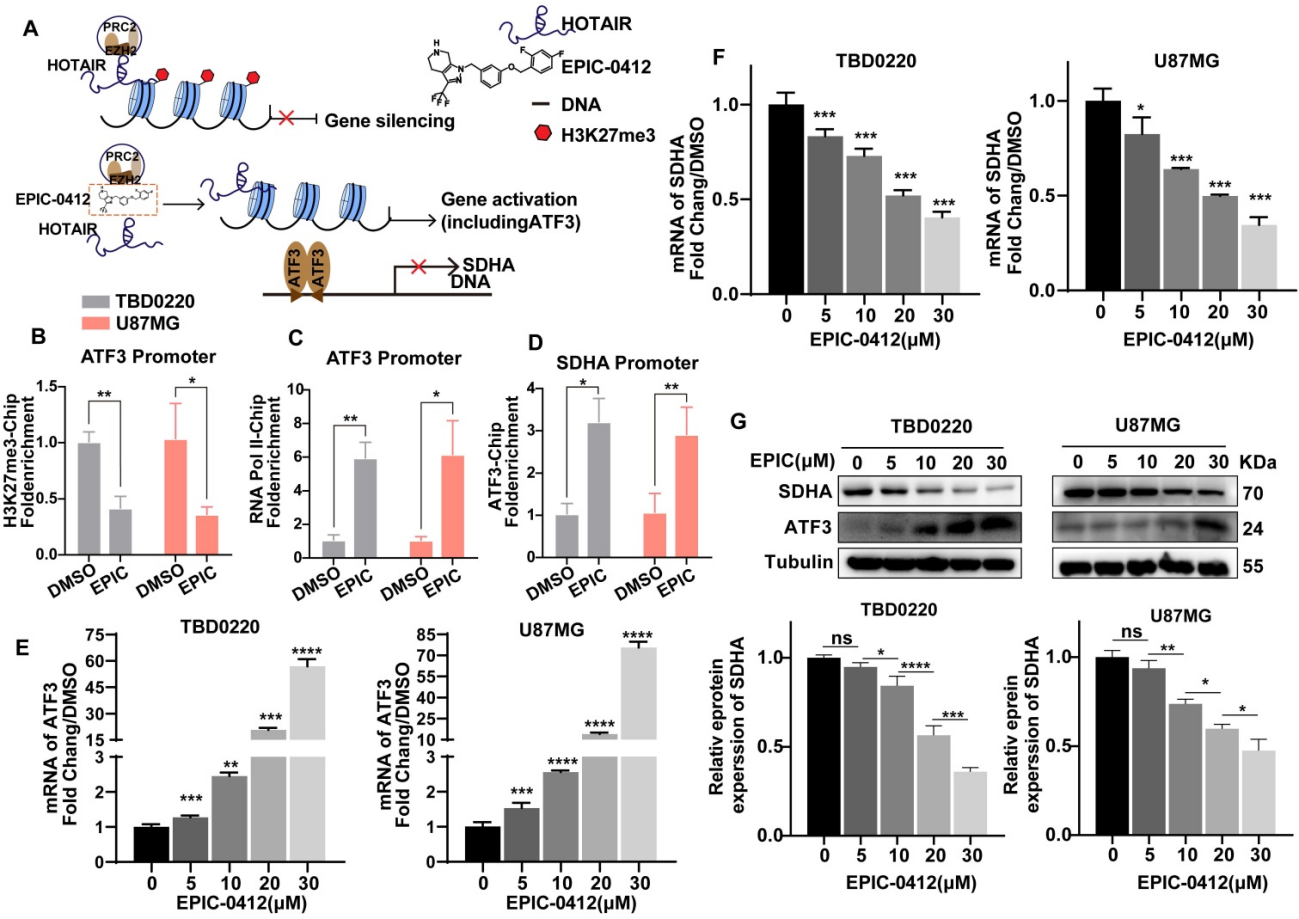
** HOTAIR-ATF3-SDHA axis accounts for deteriorated TCA cycle under EPIC treatment. (A)** Mechanism of action of EPIC and subsequent downstream epigenetic phenotypic changes.** (B-C)** ChIP assay showing the binding of H3K27me3 at the promoters of ATF3 **(B)**, and RNA polymerase II **(C)**, after treatment with 20 µM of EPIC for 48 h (n = 3).** (D)** ChIP assay at the SDHA promoter using an anti-ATF3 antibody (n = 3). **(E-F)** Relative mRNA levels of ATF3 **(E)** and SDHA** (F)** were measured by qRT-PCR assay after treating TBD0220 or U87MG cells with indicated concentrations of EPIC (20 µM) for 48 h (n = 3). **(G)** Representative western blot showing the expression of ATF3 and SDHA after treatment with indicated concentrations of EPIC, and relative levels of SDHA protein in TBD0220 and U87MG cells (n = 3). All data are shown as the mean values ± SD, *p* values are based on one-way or two-way ANOVA. ****p< 0.0001, ***p < 0.001, **p < 0.01, *p < 0.05; ns, nonsignificant.

Owing to the competitive binding of EPIC to the secondary structure within lncRNA HOTAIR that is recognized by EZH2, we successfully blocked the PRC2 recruitment and remodeled the trimethylation level of H3K27 to prompt the expression of tumor suppressor genes (**Figure 2A**). To further clarify the effect of EPIC on tumor suppressors' expressions, we performed the ceRNA microarray assay to reveal the differences in gene expression patterns after EPIC treatment. Notably, our results showed that a total of 444 genes were upregulated, while 293 genes were downregulated after EPIC treatment in ceRNA experiments. The gene profiling data are displayed in **Figure S2A**, in which ATF3 was significantly elevated in response to the EPIC treatment. Transient transfection and *in vitro* transcription assays indicated that the longer isoform of ATF3 was directed to homodimerize and suppress the promoter activation 35, 36.

To examine that ATF3 was significantly induced due to the low level of H3K27 methylation modulated by EPIC, we performed a ChIP assay to revalidate the results described before. Consistently, we observed a lower occupancy of H3K27me3 (**Figure 2B**) at the ATF3 promoter locus but with a higher binding to RNA polII (**Figure 2C**), indicating a persistent transcriptional activation. Although contents of TCA cycle metabolites like citric acid, aconitic acid, isocratic acid, α-oxoglutaric acid, and fumaric acid were significantly decreased, however, the succinic acid level did not alter following the EPIC treatment (**Figure 1D**). Thus, we focused on the enzymes that could convert succinate to fumarate, and we found that SDHA, rather than SDHB, SDHC, or SDHD was regulated by EPIC (**Figure S2B**). Further analysis of the TCGA GBM dataset revealed a negative correlation between ATF3 and SDHA gene expressions (**Figure S2C**). The ChIP assay was performed with an anti-ATF3 antibody which indicated positive binding of ATF3 to the SDHA promoter (**Figure 2D**). ATF3 binding on the SDHA promoter was increased in DMSO-treated cells. Moreover, EPIC treatment significantly stimulated this binding, suggesting an intrinsic binding affinity of ATF3 for the SDHA promoter. Consistent with the result that ATF3 repressed the SDHA transcription by blocking its promoter activation, both the SDHA mRNA and protein levels were decreased following EPIC stimulation in both TBD0220 and U87MG cells in comparisons to respective mock-treated cells (**Figure 2E-G),** indicating that the expressions of ATF3 and SDHA had a dose-dependent effect for EPIC. To analyze whether ATF3 was required for the repression of SDHA transcription, we measured the SDHA transcript level by qRT-PCR and protein level by WB in ATF3 downregulated cells. ATF3 depletions in both TBD0220 and U87MG cell lines increased the expression of SDHA in transcript and protein levels (**Figure S2D-E**). Furthermore, there were no significant changes in the mRNA expressions of ATF3 and SDHA in ATF3 downregulated cells with or without EPIC exposure (**Figure S2F**). To demonstrate the direct regulatory role of ATF3 on the SDHA expression profile, we performed the ChIP assay using an anti-ATF3 antibody in ATF3 knockdown cells. The results showed a loss of ATF3 occupancy at the promotor region of the SDHA gene following the ATF3 knockdown, whereas the ATF3 level did not change when ATF3 was knocked down in cells treated with EPIC (**Figure S2G**). Collectively, ATF3-regulated SDHA expression could be repressed by EPIC, resulting in mitochondrial respiration disruption and altered metabolic responses in glioma cells.

### Reduction in ATP production hinders cell proliferation and contributes to G1/S arrest in GBM

**Figure 3:**
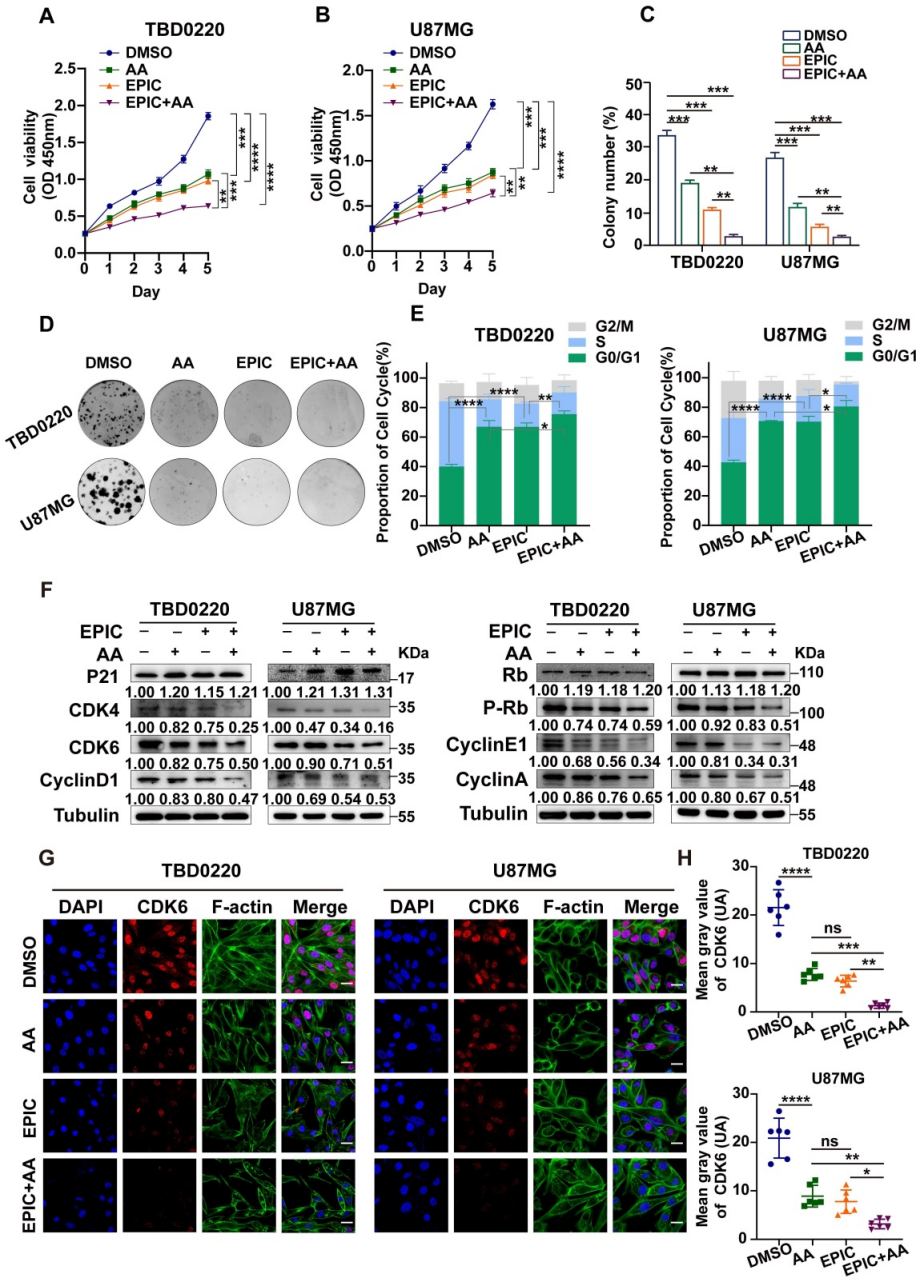
** Reduction in ATP production hinders cell proliferation and contributes to G1/S arrest in GBM. (A-B)** The relative viability of TBD0220 **(A)** and U87MG **(B)** cells was measured using a CCK-8 kit (n = 3).** (C-D)** Colony formation assay to detect GBM cell growth **(D),** and the quantification of colony numbers** (C)** (n = 3).** (E)** Cell cycle analysis using flow cytometry after incubation with different treatments like EPIC (20 µM), AA (25 µM), or EPIC (20 µM) + AA (25 µM) for 48 h, and the results are plotted as a histogram (n = 3) **(F)** Representative western blotting showing the expression of p21 and Rb and their downstream targets. The results were normalized to Tubulin with the control group as 1. Protein expression was quantified by ImageJ.** (G)** Representative confocal images of CDK6 after the treatment with AA (25 µM), EPIC (20 µM), or EPIC (20 µM) + AA (25 µM) for 48 h (n = 6). **(H)** The quantitative analysis of fluorescence images of CDK6 (n = 6). All data are shown as the mean values ± SD, and *p* values are based on one-way or two-way ANOVA. ****p < 0.0001, ***p < 0.001, **p < 0.01, *p < 0.05. Scale bar = 20 μm.

Faced with the phenotype of reduced ATP production, we observed a stark decline in the number of proliferating cells following the treatment of anti-tumor agents in comparison to vehicle-treated controls, consistent with the OCR results showing that the combination therapy of EPIC and AA was more effective than monotherapy of EPIC or AA in TBD0220 (**Figure 3A**) and U87MG (**Figure 3B**) cells. Furthermore, the colony formation assay exhibited a consistent result with CCK-8 assay that the number of colonies changed after treatment with different therapies, and the combination group had a stronger inhibitory effect on the cell proliferation than the monotherapy (**Figure 3C-D**).

To reveal the underlying mechanism of ATP content-associated modulation of cell proliferation rate, we measured the cell cycle status using flow cytometry. Glioma cells incubated with EPIC, AA, or EPIC+AA for 48h showed a significantly increased number of cells in the G0/G1 phase of the cell cycle (**Figure 3E**). In other words, the inhibition of ATP production blocked the synchronized cell cycle progression into the G0/G1 phase arrest. Besides, dual therapy of EPIC and AA showed more obvious changes in the cell cycle distribution pattern than monotherapy. Previous studies have demonstrated that p21 blocks the activity of cyclin-CDKs (cyclin-dependent kinase) complex preventing cell cycle progression from G1 to S phase 37. Detection of the G0/G1 phase-specific checkpoint markers by WB revealed that treatment with either AA or EPIC increased the protein level of p21 and decreased its downstream proteins, such as cyclin D1, CDK4, and CDK6, while the combined treatment showed a higher level of alteration of these proteins (**Figure 3F, left**). Cyclin D1, CDK4, and CDK6 formed an active kinase complex that phosphorylated the retinoblastoma protein (RB) 38-40. Hence, we evaluated levels of Rb, p-Rb, cyclin E1, as well as cyclin A following the above treatments. WB indicated that the protein levels of p-Rb, cyclin E1, and cyclin A were reduced, while the level of Rb was increased (**Figure 3F, right**). Consistent with the results of flow cytometry analysis, the combination treatment (EPIC + AA resulted in a significant regulation of related protein levels. Then, IF analysis was performed to confirm CDK6 protein level after treatment with EPIC or/and AA in TBD0220 and U87MG cells. As shown, compared with the DMSO-treated control cells, monotherapy of EPIC or AA in GBM cells revealed significantly lighter fluorescence intensities, and this effect was intensified by several folds following the combination treatment (**Figure 3G**). Quantitative analysis of the CDK6 fluorescence intensities across the groups showed that the EPIC + AA group had the lowest level of CDK6 than the other groups (**Figure 3H**).

To further verify that the inhibition of ATP synthesis led to the G0/G1 arrest by energy loss, we added ATP to reverse these therapy-induced G0/G1 arrests. As illustrated in **Figure S3A**, flow cytometry showed that supplementation with ATP partly rescued the G0/G1 phase arrest caused by the treatments (EPIC, AA, EPIC + AA) in glioma cells. Consistently, the addition of ATP partially reversed the increase in p21 level and decrease in cyclin D1, CDK4, and CDK6 levels. Moreover, p-Rb and its downstream proteins were increased (**Figure S3B-C**). Also, we observed that the addition of ATP partially rescued cells from the inhibited proliferation (**Figure S3D**). Based on these results, we speculated that ATP storage might play an important role in mediating GBM cell cycle progression and proliferation. Taken together, these data demonstrate that ATP storage is essential for GBM cell cycle regulation, and the lack of it cripples cell proliferation deeply.

### The RTK-RAS pathway contributes to TCA cycle modulation and ATP production in GBM

**Figure 4:**
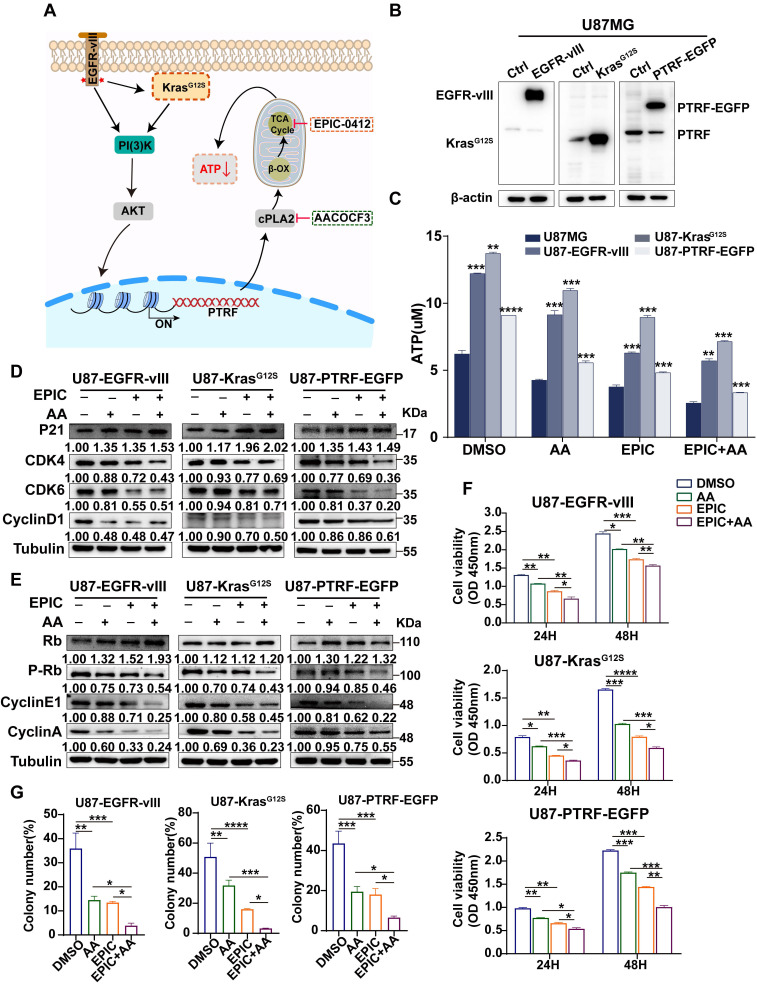
** The RTK-RAS pathway contributes to TCA cycle modulation and ATP production in GBM. (A)** The proposed mechanistic scheme by which the RTK-RAS pathway reprograms the energy metabolism in GBM.** (B)** Representative western blotting showing the overexpression of EGFR-vIII, mutant Kras^G12S^, and PTRF-EGFP in U87MG cells (cells transduced with empty vector or corresponding lentivirus). **(C)** Direct measurement of the intracellular total ATP concentration by bioluminescence after the treatment with AA (25 µM), EPIC (20 µM), or EPIC (20 µM) + AA (25 µM) for 48 h (n = 3). **(D-E)** Representative western blotting showing the expression of p21 and Rb and their downstream targets after the treatment with AA (25 µM), EPIC (20 µM), or EPIC (20 µM) + AA (25 µM) for 48 h (n = 3). The results were normalized to Tubulin with the control group as 1. Protein expression was quantified by ImageJ. **(F-G)** Cell viability and colony formation assays were performed, and the numbers of colonies were calculated (n = 3). All data are shown as the mean values ± SD, and *p* values are based on one-way or two-way ANOVA. ****p< 0.0001, ***p < 0.001, **p < 0.01, *p < 0.05.

Studies have shown that in the receptor tyrosine kinase (RTK)-RAS pathway, irrespective of tumor types, Kirsten rat sarcoma* (KRAS)* is the most frequently altered gene, followed by *BRAF,* and *EGFR* 41. KRAS activates the PI3K-AKT axis as well as the RAF-MEK-ERK (MAPK) kinase cascade 42. In GBM, 86% of samples harbor a minimum of one genetic event within the RTK/PI3K pathway 43, indicating that RTK/RAS/PI3K signaling pathway competes for an important role in tumorigenesis. The variant III (vIII) deletion of the extracellular domain (vIII mutant) activating *EGFR* expression has been the most commonly observed phenotype in GBM 43. PTRF is upregulated by the activation EGFR/PI3K/AKT signaling pathway 29, remodeling the membrane phospholipid composition 12. The therapies with EPIC or/and AA were performed in glioma cells in this study (**Figure 4A**). Accordingly, EGFR-vIII, mutant KrasG12S, and PTRF-EGFP expressing lentiviral vectors were transduced into the U87MG cells to mimic the sustained activation of the RTK/RAS/PI3K signaling pathway, and the corresponding protein expressions were measured (**Figure 4B**). Compared to respective control cells, ATP concentrations were significantly increased in GBM cells after transduction, and mutant KrasG12S expression showed higher efficiency in elevating ATP production, followed by EGFR-vIII, and PTRF-EGFP (**Figure 4C**). To confirm whether the lncRNA HOTAIR-EZH2 interaction blocker EPIC and cPLA2 inhibitor AA could reduce ATP production in a more malignant GBM model, we repeated the ATP concentration measurement in cells with monotherapy or combination therapy. Interestingly, ATP concentrations were significantly decreased following the inhibitor treatment, and the combination of EPIC and AA was more effective than the monotherapy (**Figure 4C**), which were aligned with results in TBD0220 and U87MG cells.

As was identified before, reduction in ATP production contributed to transforming tumor cells into the cell-cycle arrest stage. We then evaluated expressions of cell cycle-related proteins, including p21, CDK4, CDK6, cyclin D1, Rb, p-Rb, cyclin E1, and cyclin A. WB showed that in addition to the increased Rb level and levels of p21, CDK4, CDK6, cyclin D1, Rb, p-Rb, cyclin E1, and cyclin A were also decreased. Furthermore, the expression of proteins in cells with combination therapy was significantly reduced compared to that of monotherapy, suggesting that this therapy could exert its effectiveness even though the RTK/RAS signaling pathway is activated (**Figure 4D-E**). To examine the proliferation of the malignant glioma cells, we further carried out a cell viability assay (**Figure 4F**), and colony formation assay (**Figure 4G, Figure S4A**). Collectively, we conclude that not only the monotherapy of EPIC or AA but also the combination therapy could highly repress the proliferation of GBM cells. The results showed despite the activation of the RTK-RAS-PI3K signaling pathway stimulating the TCA cycle, this targeted inhibition of ATP synthesis could efficiently inhibit the growth and proliferation of glioma cells in the context of energy metabolism.

### Disruption of phospholipid metabolism and the TCA cycle suppresses tumor growth and prolongs survival *in vivo*

**Figure 5:**
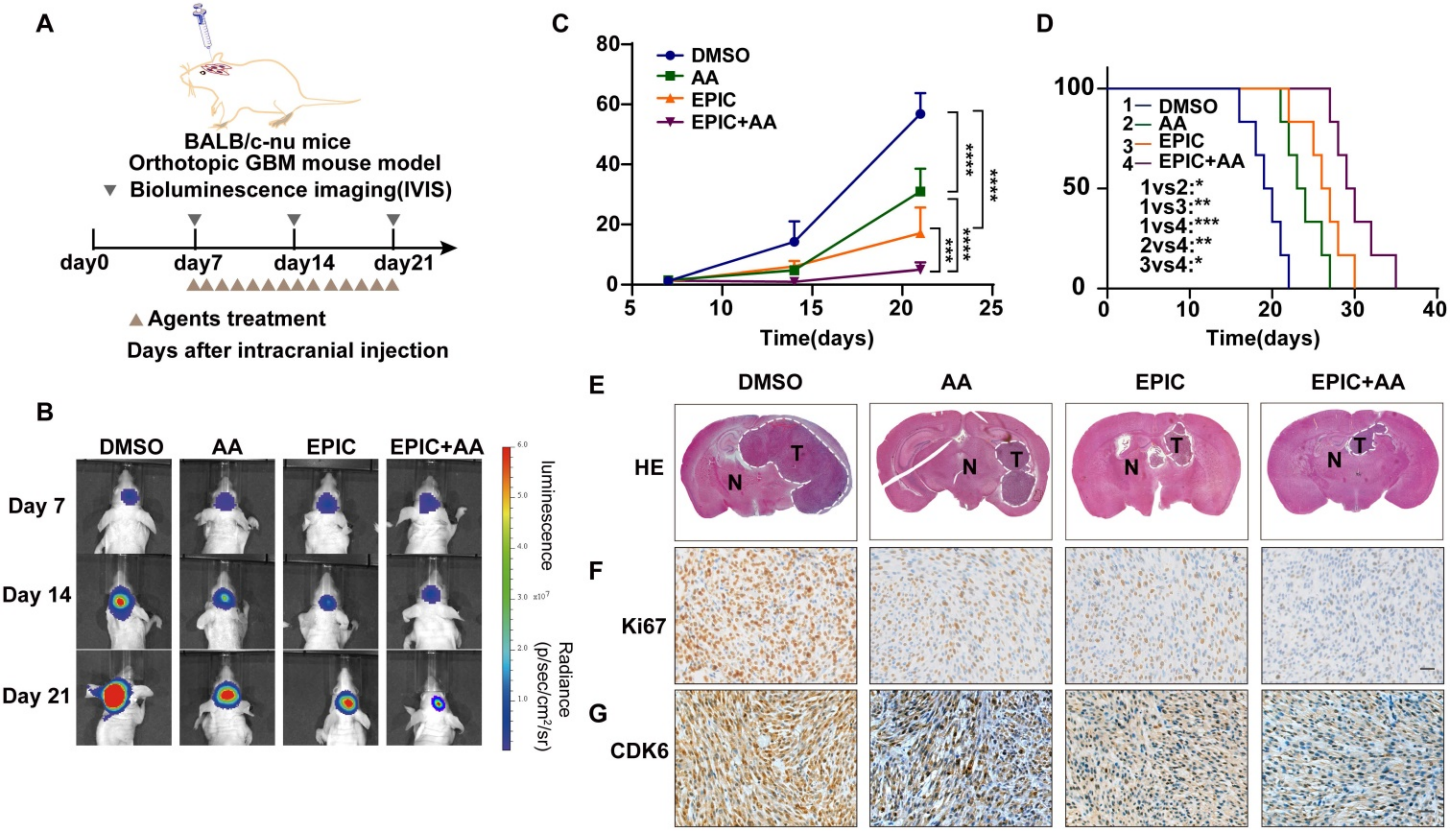
** Disruption of phospholipid metabolism and the TCA cycle suppresses tumor growth and prolongs survival *in vivo*. (A)** Schematic of the patient-derived GBM intracranial xenograft model in BALB/c nude mice (n = 7 for each group). **(B)** Representative tumor bioluminescence images of mice from four groups DMSO-treated, AA-treated (25 mg/kg), EPIC-treated (15 mg/kg), and EPIC (15 mg/kg) + AA-treated (25 mg/kg) mice at 7, 14, and 21 days after tumor implantation. **(C)** Quantification of bioluminescence imaging signal intensities from all groups (n = 6-7). **(D)** Survival rates of mice with indicated treatments are exhibited by the Kaplan-Meier survival plot (n = 6-7). **(E)** Representative images of H&E staining showing tumor volume in the nude mice (T denotes tumor, N denotes normal brain tissue). Images were collected on an Olympus instrument and are displayed at a 10× magnification. **(F-G)** Representative images of IHC staining for Ki67 and CDK6 in tumor tissues from patient-derived xenograft models. All data are shown as the mean values ± SD, and *p* values are based on one-way ANOVA. ****p< 0.0001, ***p < 0.001, **p < 0.01, *p < 0.05. Scale bar = 50 μm.

To identify whether the fatty acid metabolism and mitochondrial respiration play critical roles in sustaining GBM growth *in vivo*, intracranial tumor models were established in female BALB/c nude mice by exploiting the patient-derived TBD0220 cells expressing luciferase reporter to propel non-invasive tumor monitoring in mice brains (**Figure 5A**). When tumors in the brain were detectable, we grouped mice randomly and treated them with either monotherapy or combination therapy of EPIC and AA. Bioluminescence imaging analysis exhibited that the monotherapy of EPIC or AA resulted in a significant regression in tumor growth, while the combination therapy had a more striking suppression effect on tumor growth (**Figure 5B-C**). Moreover, the tumor-bearing mice with a low energy metabolism had a longer survival as seen in the Kaplan-Meier survival curve. Compared with the DMSO control, the median survival time rose from 19.5 days to 23.5 days when AA was used; from 19.5 days to 26.5 days when EPIC was used; and from 19.5 days to 29.5 days when the dual therapy was employed, showing an approximately 51% increase in survival rate than DMSO group (**Figure 5D**). Additionally, the combination therapy mice group showed an approximately 25.5% increase in the survival rate over the AA monotherapy mice group and an approximately 11.3% increase in the survival rate of the EPIC monotherapy mice group (**Figure 5D**). H&E staining of brain tumor tissue sections revealed an obvious decrease in tumor volume in groups with effective treatments in contrast to the control group (**Figure 5E**). Additionally, IHC staining indicated that GBM cells suffered from a massive hindrance in proliferation, which was reflected by the decreased levels of Ki67 (**Figure 5F**), and CDK6 (**Figure 5G**). Consistently, the dual therapy group exerted a stronger tumor inhibition than monotherapy, according to the H&E and IHC staining. Furthermore, we performed the blood routine and liver and kidney function tests in mice following repeated administrations of EPIC, AA or EPIC + AA at the 2^nd^ or 4^th^ week. H&E staining of the major organs of mice was performed at the 4^th^-week post-treatment to assess the biosafety of repeated administrations of therapeutics. The results indicated that repeated doses of EPIC and/or AA had no significant adverse effect on normal tissues/cells (**Table S1-2, Figure S4B**). The body weights of mice were measured every two days during the 4^th^ week. At the end of the 4^th^ week, we discovered no discernible variation in body weight across the four treatment groups (**Figure S4C**). Taken together, these data demonstrate that the inhibition of ATP synthesis can prolong the survival rate of tumor-bearing mice, which might be a promising and potential therapeutic strategy for managing GBM.

### The combination therapy of EPIC and AA dose not affect glycolysis

Glycolysis is regarded as a critical supporter of tumorigenesis. Thus, to explore whether EPIC and AA had any impact on glycolysis, we determined the rate of glycolysis by quantifying PER, which suggested bulk acidification (**Figure S5A-5B**). As illustrated, compared with the DMSO group, both basal glycolysis (**Figure S5C-D**) and compensatory glycolysis (**Figure S5E-F**) had no difference between the EPIC monotherapy and the combination therapy groups but displayed an accelerated glycolysis rate in the AA therapy group, that were consistent in TBD0220 and U87MG cells. These results reflected changes in the lactate accumulations under different treatments. Furthermore, we found that the difference in the rates of lactate production between the combination therapy group and the DMSO control group concurred with the glycolysis rate data (**Figure S5G**). HK2 is a vital rate-limiting enzyme in glycolysis and is usually overexpressed in cancer cells, resulting in activated glycolysis 44. WB analysis (**Figure S5H**) revealed that the expression of HK2 was steady even when treated with different inhibitors. The reason why the AA monotherapy group could induce changes in glycolysis remains unclear. But we conducted a non-targeted metabolic analysis to detect the change of metabolism in TBD0220 cells after treatment with AA. Metabolic pathway enrichment showed that the ferroptosis pathway was drastically affected (**Figure S5I**), which might be relative to the compensatory glycolysis. In summary, the glycolysis of glioma cells was not influenced by the combination therapy of EPIC and AA.

### Targeting the TCA-phospholipid-glycolysis axis by triple therapy in GBM leads to growth inhibition *in vitro* and *in vivo*

**Figure 6:**
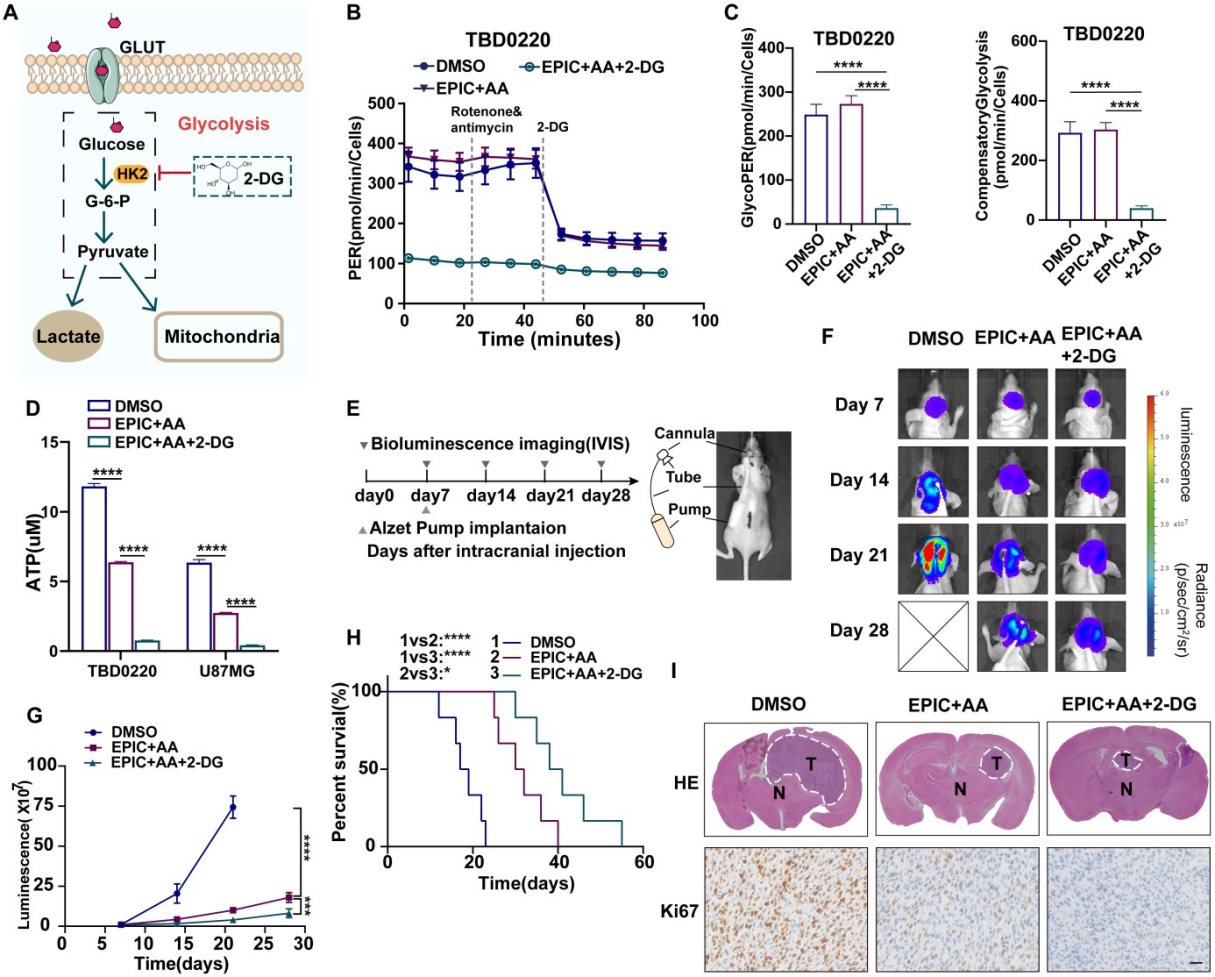
** Targeting the TCA-phospholipid-glycolysis axis by triple therapy in GBM leads to growth inhibition *in vitro* and *in vivo*. (A)** HK2-mediated glycolysis could be disrupted by 2-DG. **(B)** PER was measured by glycolysis rate kit on a Seahorse Analyzer in TBD0220 cells after the treatment with DMSO, EPIC (20 µM) + AA (25 µM) or EPIC (20 µM) + AA (25 µM) + 2-DG (50 mM) for 24 h (n = 3-4 replicates per group). **(C)** The measurement of glycolytic proton efflux rate (glycoPER), namely basal glycolysis, and compensatory glycolysis rate in TBD0220 cells (n = 3-4). **(D)** Measurements of the intracellular total ATP concentrations in TBD0220 and U87MG cells (n = 3). **(E)** CDE with Alzet micro-osmotic pumps was implanted under the skin of tumor-bearing mice (n = 7 for each group). **(F)** Representative bioluminescence images of intracerebral cancer mice that were treated with DMSO, the dual therapy of AA (25 mg/kg) and EPIC (15 mg/kg), or the triple therapy of AA (25 mg/kg), EPIC (15 mg/kg), and 2-DG (15 mg/kg) at days 7, 14, 21, and 28 post-implantation. **(G)** Quantitation of bioluminescence signals (n = 6-7). **(H)** Kaplan-Meier survival curve shows the overall survival of mice in (E) (n = 6-7). **(I)** Representative brain images from mice with indicated treatments and corresponding H&E (T denotes tumor, N denotes normal brain tissue) and IHC staining images of Ki67. All data are shown as the mean values ± SD, and p values are based on one-way or two-way ANOVA. ****p< 0.0001, ***p < 0.001, *p < 0.05. Scale bar = 50μm.

As described above, glycolysis was not modulated by our combination therapy of EPIC and AA, which mainly disrupted the TCA cycle and lipid metabolism. Thus, to block glycolysis, we used 2-DG, a selective inhibitor of HK2 (**Figure 6A**). After treating TBD0220 cells with dual therapy (EPIC and AA), and triple therapy (EPIC, AA, and 2-DG) for 24 h, respectively, glycolysis rates were measured to quantify PER (**Figure 6B**), which revealed that basal glycolysis rate in triple therapy group was 85% lower than that of the control group, and 87% lower than that of the dual therapy group. The compensatory glycolysis rate in the triple therapy group was 86% lower than that of the control group and 87% lower than the dual therapy group (**Figure 6C**). These differences were in agreement with the glycolysis data measured in U87MG cells and demonstrated an almost 90% reduction among the three groups (**Figure S6A-B**). Furthermore, the total intracellular ATP concentrations were evaluated, and the ATP content in the triple therapy group was reduced in contrast with the dual therapy and DMSO groups (**Figure 6D**). Moreover, in TBD0220 and U87MG cells, ATP levels were decreased by 90% and 95%, respectively. We found that lactate production was significantly reduced following the triple therapy (**Figure S6C**).

Since ATP is critically required for anabolic and metabolic processes in cancer cells, we performed a CCK-8 proliferation assay (**Figure S6D**) and a colony formation assay (**Figure S6E**) to investigate the inhibitory effects of anti-tumor agents on cell proliferation. The cell proliferation assay with the TBD0220 cell line indicated that, in contrast with control cells, the dual therapy resulted in an obvious decrease in the proliferation rate, which was higher than the triple therapy group. TBD0220 cells treated with triple therapy, including EPIC, AA, and 2-DG, showed a lower number of colonies than those in dual therapy (EPIC and AA) and control groups. Consistent results were also observed in U87MG cells.

Given that the RTK-RAS pathway also regulates the energy metabolism, we measured the intracellular ATP concentration and cell proliferation by CCK-8 and colony formation assays in U87MG cells overexpressing EGFR-vIII, mutant Kras^G12S^, or PTRF-EGFP, respectively. The ATP concentrations were decreased in these cells with triple therapy and were more significant than the dual therapy (**Figure S6F**). ATP measurements revealed that ATP concentrations of U87MG cells overexpressing EGFR-vIII, mutant Kras^G12S^, and PTRF-EGFP in the triple therapy group were 71%, 74%, and 70% lower than the control group, and 47%,45%, and 49% lower than the dual therapy group, respectively. Consistently, in the colony formation (**Figure S6G**), and CCK-8 assays (**Figure S6H**), the treatment groups with the triple and dual therapies exhibited a significantly decreased growth in comparison to respective control groups.

The blood-brain barrier (BBB) is regarded as the main obstacle in hindering the vascular delivery of a variety of therapeutic substances to brain tumors 45. Thus, to further assess the therapeutic efficiency in the GBM model *in vivo*, we conducted direct cerebral intra-tumoral delivery of the drugs at effective levels determined by CED via a micro-osmotic pump bypassing the BBB. Micro-osmotic pumps were implanted under the skin and were stereotactically placed in intracerebral tumor locations in tumor-bearing mice (**Figure 6E**). The delivery of drugs via micro-osmotic pumps contained enough treatment solutions, including DMSO vehicles, dual therapy (EPIC and AA), and triple therapy substances (EPIC, AA and 2-DG). Tumor progression was detected by bioluminescence imaging (**Figure 6F**), and the results indicated not only the administration of dual therapy but also triple therapy could contribute to a significant decrease in tumor growth in comparison to the DMSO vehicle-treated group. The triple therapy exhibited a smaller lesion area and weaker intensities in bioluminescence assays in contrast to the dual therapy, suggesting a stronger therapeutic response to GBM (**Figure 6G**). The delivery of triple and dual therapy substrates to intracerebral tumors in animals increased the overall survival in comparison to the vehicle control mice, as seen in Kaplan-Meier survival curves. Compared with the DMSO control, the median period of survival of brain tumor-bearing mice rose from 18 days to 39.5 days by the intra-tumoral delivery of a triple therapeutic regimen, suggesting an approximately 119% increase in the survival rate. Intra-tumoral delivery of dual drugs also increased the median survival time from 18 days to 31 days, showing an approximately 72% increase in the survival rate. Importantly, the triple therapy mice group showed approximately 27.4% of the increase in the survival rate than the dual therapy mice group (**Figure 6H**). H&E staining also exhibited that mice with treatment showed reduced tumor volume and cellular density in comparison to mice treated with vehicle control (**Figure 6I**). We also tested Ki67 positivity among three groups with triple therapy or dual therapy and paired with the control group by IHC staining, which showed there were fewer Ki67 positive cells in drugged tumors compared with vehicle-treated tumors, suggesting potential tumor suppression. Furthermore, tumor volume, cellular density, and proliferation rates were decreased significantly in the triple therapy group than in the dual therapy group (**Figure 6I**). To further evaluate the toxicity of triple therapy, the liver and kidney functions and blood routine were examined at the end of the 2^nd^ and 4^th^ week, and the vital organs of mice like heart, liver, spleen, kidney, and lung were collected for H&E staining at the end of 4^th^ week and body weights were recorded every two days in the DMSO, dual therapy, and triple therapy groups. No obvious organ toxicity was observed when EPIC + AA was administered w or w/o 2-DG, compared with that of the control group mice (**Table S1-2, Figure S6I**). No discernable variations in body weights were observed across all the therapy groups (**Figure S6J**).

Taken together, these results demonstrated that the disruption of glycolysis and phospholipid metabolism, along with the TCA cycle, delayed tumor proliferation and prolonged the median survival time in mice with intracranial GBMs.

### The energy metabolism profile of the TCA-phospholipid-glycolysis axis of metabolism in GBM

**Figure 7:**
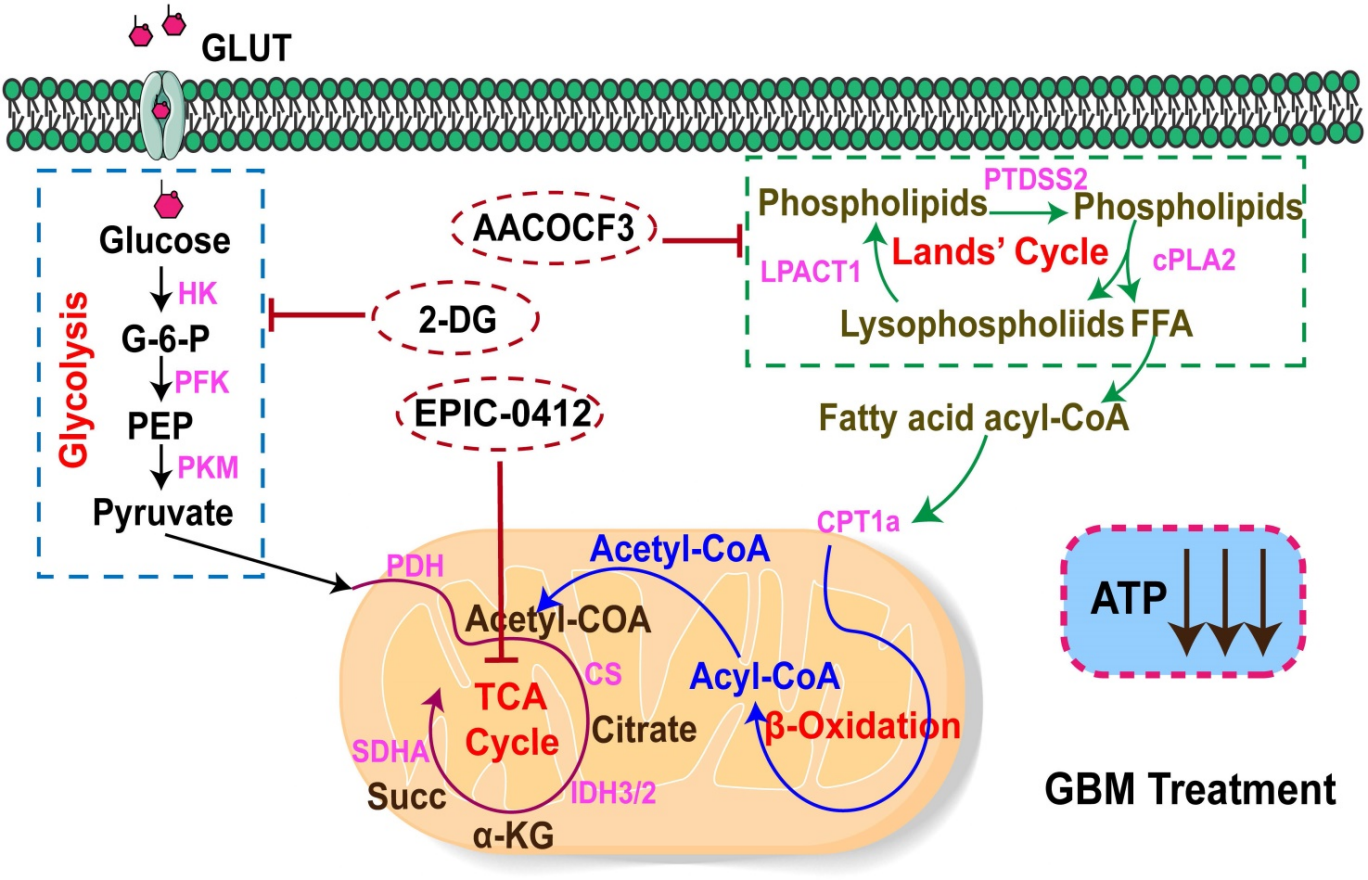
The mechanistic scheme by which the TCA-phospholipid-glycolysis metabolism pathway was blocked using specific inhibitors to suppress the tumor proliferation in GBM.

In this study, we demonstrated that there were two main metabolism pathways generating ATP to support the energy demand in GBM cells, namely the FAO and aerobic glycolysis, also called the Warburg effect **(Figure 7)**. In the fatty acid pathway, it could be possible that most FFAs accumulate from the Land's cycle, in which cPLA2 serves as a catalytic enzyme that effectively hydrolyzes phospholipids into lysophospholipids and FFAs. As a basal fuel for FAO, fatty acids are transported into mitochondria with the help of carnitine O-palmitoyl transferase 1 (CPT1A) to participate in β-oxidation and generate acyl-CoA. Moreover, glucose acts as the raw material for energy production for basic cellular activities, which is also of central importance in tumor proliferation. Warburg's effect relies on strengthening the rate-limiting enzymes, such as HK, PFK, and PKM, to fasten glycolysis, but with a low production rate. HK2 as a rate-limiting enzyme of the glycolytic pathway, plays a pivotal role in converting glucose to glucose-6-phosphate 46. Also, a fraction of pyruvates from the glycolysis, and acyl-CoA from β-oxidation could both be converted into acetyl-CoA to support the TCA cycle for ATP production in tumor cells.

Therefore, we first proposed a therapeutic scheme by which the crucial TCA-phospholipid-glycolysis metabolism axis was blocked by tumor proliferation inhibitors. More specifically, EPIC, as a novel derivative of AQB, inhibited SDHA expression and slowed the TCA cycle turnover, thereby suppressing ATP synthesis. Mechanistically, AA, an effective inhibitor of cPLA2, could serve as a hydrolase for generating FFAs to suppress FAO. While 2-DG acts as a competitive inhibitor in the glucose metabolism pathway, inhibiting glycolysis via its action on HK2 in tumors. Our findings thus identified that the checkpoints of the TCA, phospholipid, and glycolysis pathways were SDHA, cPLA2, and HK2, respectively. The triple therapy with EPIC, AA, and 2-DG was specifically pointed to these three checkpoints, which significantly decreased the ATP production and dramatically hindered tumor growth in the GBM model.

## Discussion

In this study, we proposed a mechanistic scheme by which the TCA-phospholipid-glycolysis metabolism axis could be blocked by specific inhibitors to dramatically hinder GBM cell proliferation. This is the first study exploring the impairment of ATP production for anti-tumor effects. Cancer cell metabolic reprogramming to meet the energy requirement is required for tumor progression and survival 33, and ATP is the central mediator of metabolism and signaling in cells 5, 7, 47. The TCA cycle usually is fueled by metabolic intermediates generated by other metabolic pathways, such as FAO and aerobic glycolysis. GBM strengthens the FAO pathway for the high ATP production and anabolic demands of tumor cells 48. Moreover, mitochondrial ATP is required for fatty acid uptake and transport in endothelial cells 49. Specifically, the elevation of ATP contents in brain tumors results in a rapid growth rate and depressed immune responses 12. Consistently, our findings showed that because of the reduction of ATP production, the cell cycle was arrested in the G0/G1 phase and the growth rate of GBM was decreased, which was consistent with previous studies that suggested a reduction in ATP level downregulated cyclin D1 level, accounting for the G0/G1 arrest in tumor cells 50, 51.

Excessive reactive oxygen species (ROS) production and hyperinflammation are detrimental consequences of continuous SDH activity. To decrease this inflammatory response, macrophages tend to use itaconate to inhibit SDH function and the proinflammatory responses 52. In head and neck squamous cell carcinoma (HNSCC), the expression of SDHA is higher in the tumor tissues than in the tissue-matched normal mucosa, predicting a higher locoregional recurrence 53. Our study showed that the increased ATF3 level could repress the TCA cycle-related gene *SDHA*'s expression, leading to a reduced mitochondrial respiration rate and ATP contents. Previous studies have shown that ATF3 plays an anti-tumor function, and the overexpression of ATF3 reduces the migration and proliferation capacities of metastatic glioma cells 54. Activation of the ATF4‐ATF3‐CHOP axis leads to apoptosis and cell cycle arrest in GBM cells 55. In the present study, we identified that ATF3 gene transcription was regulated by H3K27me3 in association with lncRNA HOTAIR. The H3K27me3-ATF3-SDHA axis was confirmed to perturb the TCA cycle under EPIC treatment in our study, indicating that epigenetic inheritance might play an important role in tumor cells' metabolic reprogramming.

Besides, FFAs from the Land's cycle regulated by cPLA2 support energy production via the mitochondrial FAO pathway 56. Fatty acids depleted by AA, a cPLA2 inhibitor, led to a drop in energy production. Hence, the reason for the upregulated glycolytic response to AA treatment in glioma cells remains unclear. We observed that the metabolic enrichment pathway analysis of significantly altered metabolites included ferroptosis as one of the most affected metabolic pathways. AA might interfere with ferroptosis by affecting fatty acid metabolism. Remarkably, as previous studies reported, tumor cells could activate glycolysis to inhibit ferroptosis for self-preservation 57, 58. Therefore, we speculated that the increase of compensatory glycolysis might be relative to the inhibited ferroptosis. More reasons for the phenotypic variations need to be figured out in future work.

In this context, the combination therapy against different metabolic pathways provided a new window of opportunity in treating GBM. Generally, the TCA cycle integrated with OXPHOS in mitochondria generates ten ATP molecules per round, whereas glycolysis produces a net of two ATP molecules from each glucose molecule. And one molecule of palmitic acid can be completely oxidized to generate more than one hundred molecules of ATP. Subsequently, we clarified that in GBM, the rates of ATP production from FAO and TCA cycles respectively accounted for 45%, and 47% of mitochondrial respiration, whereas ATP production from glycolysis accounted for 45% of the total ATP content. In this study, the dual therapy with small molecule inhibitor EPIC and cPLA2 inhibitor AA showed a decrease of 70% in mitochondrial respiration and 50% in total ATP production. In contrast, the triple therapy containing HK2 inhibitor 2-DG, EPIC, and AA significantly reduced ATP production to 95% of total capacity. Given the powerful anticancer effect of triple therapy, we concluded that it could exert cell proliferation inhibition effects by dramatically reducing ATP production in GBM. Notably, the combination of the partial FAO inhibitor, ranolazine (Rano), and the glycolytic inhibitor, dichloroacetate (DCA), can significantly block tumor growth and increase the median survival of orthotopic CT2A and GL261 syngeneic murine models 14. In some studies, the blunted glycolysis caused by aurora kinase A (AURKA) inhibitor or histone deacetylase (HDAC) inhibitors led to the engagement of OXPHOS driven by elevated FAO 16, 59. Thus, the addition of an FAO inhibitor was necessary for further inhibiting the GBM cell proliferation. On the other hand, biochanin A (BCA), a flavonoid phenolic compound, partly impairs glycolysis and mitochondrial respiration in glioma cells 15. The triple combination of HK2-antisense oligonucleotide (ASO1), OXPHOS inhibitor diphenyleneiodonium (DPI), and FAO inhibitor perhexiline prevents the growth of multiple myeloma tumor xenografts and can achieve synthetic lethality in tumor cells 60. Therefore, the combination strategy of hindering GBM ATP levels through the inhibition of associated metabolic pathways denotes a highly promising and powerful therapeutic approach to GBM management.

Successful delivery of drugs is the most challenging issue in brain cancer therapy. The brain tumor is one of the most difficult tumors to treat, probably owing to its location in the most distinct part of the human body - the central nervous system (CNS). A distinct system of brain and blood vessel cells separates and safeguards the CNS, and it also prevents most of the bloodborne medicines from entering the milieu around brain tumors 61, 62. Currently, to cross the BBB more effectively, various types of nanoparticles, such as hyperbranched polymers, linear polymers, liposomes, dendrimers, and micelles, have been synthesized and successfully tested as carriers for brain-specific drug delivery 63. Additionally, the CED is a direct infusion technique for delivering drugs to the brain, consequently, targeted inhibitors could apply to GBM through direct administration to tumors and bypass the BBB 64. In diffuse intrinsic pontine glioma, CED could overcome the delivery barrier and accumulate pharmacological concentrations of anticancer agents within the tumor 65. Consistently, direct intra-tumoral delivery of our triple combination drugs was conducted by a micro-osmotic pump. Intracerebral infusion of anti-tumor agents was a chronic process, constantly and locally delivering agents into specific tumor regions in the mice's brains, thus improving efficacy and reducing potential systemic toxicity. In our study, CED via a micro-osmotic pump prolonged the drug retention at the fusion site and maintained an effective therapeutic dose in brain tumors.

Multiple important metabolic enzymes are modified to sustain the cancer cell growth and thus may, in turn, serve as cancer-specific therapeutic targets. Our work demonstrates that targeting metabolic enzymes (SDHA, cPLA2, HK2) with specific inhibitors (EPIC, AA and 2-DG) could extremely decrease the ATP production rate in GBM cells, leading to tumor cell proliferation inhibition. Furthermore, the CED mode of delivery of anti-tumor triple therapeutics provided a potential strategy to bypass the BBB, improving treatment outcomes and mitigating side effects. In conclusion, our study showed that the TCA-phospholipid-glycolysis targeted triple therapy could successfully disrupt the GBM energy metabolism, thus raising exciting opportunities for cancer treatments.

## Supplementary Material

Supplementary figures and tables.

## Abbreviations

GBM: Glioblastoma
ATP: adenosine triphosphate
TCA cycle: tricarboxylic acid cycle
OXPHOS: oxidative phosphorylation
SMI: small molecule inhibitor
OCR: oxygen consumption rate
PER: proton efflux rate
AACOCF3: arachidonyl trifluoromethyl ketone
2-DG: 2 deoxy-D-glucose
IB: immunoblotting
IF: immunofluorescence
CED: convection-enhanced delivery
cPLA2: cytosolic phospholipase A2
HK2: hexokinase II
FAO: fatty acid oxidation
PTRF: polymerase 1 and transcript release factor
FFA: free fatty acid
lncRNAs: Long noncoding RNAs
HOTAIR: HOX transcript antisense RNA
EZH2: zeste homolog 2
PRC2: polycomb repressive complex 2
LSD1: Lysine-Specific Demethylase 1
H3K27me3: trimethylation of histone H3 Lys27
H3K4me2: dimethylated histone H3 Lys4
ceRNA: competing endogenous RNA
ChIP: immunoprecipitation
WB: Western blotting
CLSM: Confocal laser scanning microscope
H&E: Hematoxylin and Eosin
IHC: immunohistochemistry
ATF3: activating transcription factor 3
SDHA: succinate dehydrogenase complex flavoprotein subunit A
CDK: cyclin-dependent kinase
RTK: receptor tyrosine kinase
KRAS: Kirsten rat sarcoma
vIII: variant III
RB: retinoblastoma protein
p-Rb: phosphorylated retinoblastoma protein
glyPER: lycolytic proton efflux rate
BBB: blood-brain barrier
CPT1A: carnitine O-palmitoyl transferase 1
PFK: phosphofructokinase
PKM: pyruvate kinase M
HNSCC: head and neck squamous cell carcinoma
ROS: reactive oxygen species
Rano: ranolazine
DCA: dichloroacetate
AURKA: aurora kinase A
HDAC: histone deacetylase
BCA: biochanin A
ASO: antisense oligonucleotide
DPI: diphenyleneiodonium
CNS: central nervous system
