# Supplementary material for: TCA-phospholipid-glycolysis targeted triple therapy effectively suppresses ATP production and tumor growth in glioblastoma
Source: Theranostics. 2022 Oct 3;12(16):7032–50. doi: 10.7150/thno.74197 (PMC9576613; doi:10.7150/thno.74197)
Supplement: Supplementary file 1 — Supplementary figures and tables. [file thnov12p7032s1.pdf]

Supplementary materials

Supplementary Tables

Table S1. Values of serum enzymes in blood samples of mice treating with indicated therapy after continuous treatment in indicated time.

Time	Group	ALT (IU/L)	AST (IU/L)	TBIL (μmol/L)	ALB/ GLOB	BUN (mmol/L)	CREA (μmol/L)
2th week	DMSO	58.9±1.67	168.1±24.07	2.54±1.14	28.9±2.33	11.57±2.21	27.8±2.65
	AA	54.1±7.49	163.1±13.57	1.13±0.44	24.6±1.36	8.96±1.49	23.6±1.59
	EPIC	58.7±8.33	180.7±22.54	0.98±0.18	26.2±4.61	12.97±1.75	30.1±1.96
	EPIC+AA	63.9±5.76	166.9±23.07	1.43±0.54	25.0±1.74	9.84±1.82	26.4±1.77
	EPIC+AA +2-DG	56.0±2.90	171.1±10.19	1.58±0.44	26.6±0.62	9.37±0.42	22.8±0.65
4th week	DMSO	54.5±8.47	219.6±43.07	1.06±0.89	22.35±2.71	10.33±1.07	29.1±1.38
	AA	55.2±6.95	211.1±31.95	1.40±0.28	23.20±1.82	11.92±0.07	29.7±1.97
	EPIC	56.1±7.54	205.9±46.01	1.25±0.36	22.53±1.54	11.83±0.53	27.6±1.96
	EPIC+AA	49.1±12.06	235.7±17.36	0.91±0.41	21.47±1.68	11.63±0.52	29.2±1.30
	EPIC+AA +2-DG	59.2±4.25	238.7±35.64	0.85±0.28	21.60±0.33	9.88±1.61	28.4±0.86

Table S2. Blood routine analysis in blood samples of mice treating with indicated therapy after continuous treatment in indicated time.

Time	Group	WBC (×10^9/L	RBC (IU/L)	Hbg (g/L)	Plt (×10^9/L)
2th week	DMSO	6.60±0.42	9.89±0.53	147.33±8.17	944.67±122.6
	AA	6.67±1.89	9.82±0.33	143.67±5.43	924.67±175.8
	EPIC	6.17±1.86	9.59±0.27	136.01±0.82	848.05±132.0
	EPIC+AA	6.30±1.84	9.07±0.17	130.33±2.62	862.33±116.7
	EPIC+AA +2-DG	5.30±2.78	9.52±0.11	138.50±2.94	871.50±91.1
4th week	DMSO	5.92±0.65	9.37±1.05	136.46±10.57	1098.76±223.95
	AA	6.37±0.26	9.92±0.30	139.67±7.13	1132.67±33.21
	EPIC	6.15±0.41	9.43±0.75	133.76±13.95	1067.46±218.76
	EPIC+AA	5.70±2.33	9.54±0.55	138.00±9.42	1036.30±92.63
	EPIC+AA +2-DG	5.73±0.31	9.50±0.37	138.67±6.18	1022.33±114.93

Supplementary Figures

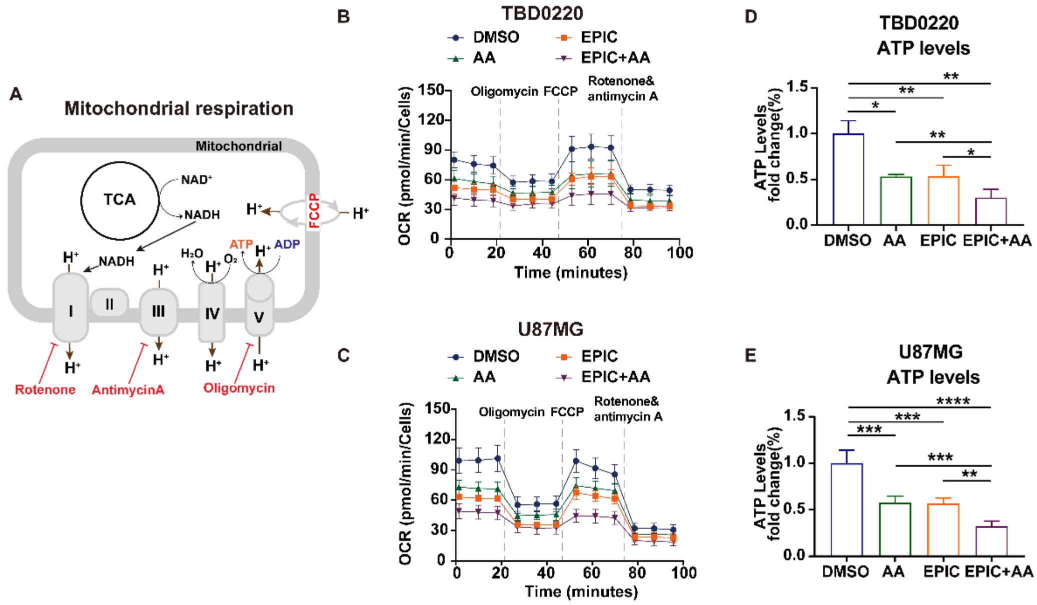

Figure S1. (A) Schematic diagram of mitochondrial stress test kit, and the target for metabolic inhibitors of oligomycin, FCCP, and rotenone/antimycin A, which shows in red. Oligomycin inhibits ATP synthase, FCCP is an uncoupler of oxidative phosphorylation (**OXPHOS**) in mitochondria, rotenone and antimycin A inhibit complex I and complex III, respectively. **(B-C)** TBD0220 **(B)** and U87MG **(C)** cells are analyzed by mitochondrial stress test on a Seahorse XFe24 Metabolic Analyzer, and the results were plotted. The addition of metabolic inhibitors is indicated (n = 3-4). **(D-E)** The FC of ATP production rate from TBD0220 **(D)** and U87MG **(E)** cells all groups (n = 3-4). All data are shown as the mean values ± SD, p values are based on one-way ANOVA. ****$p < 0.0001$, ***$p < 0.001$, **$p < 0.01$, *$p < 0.05$.

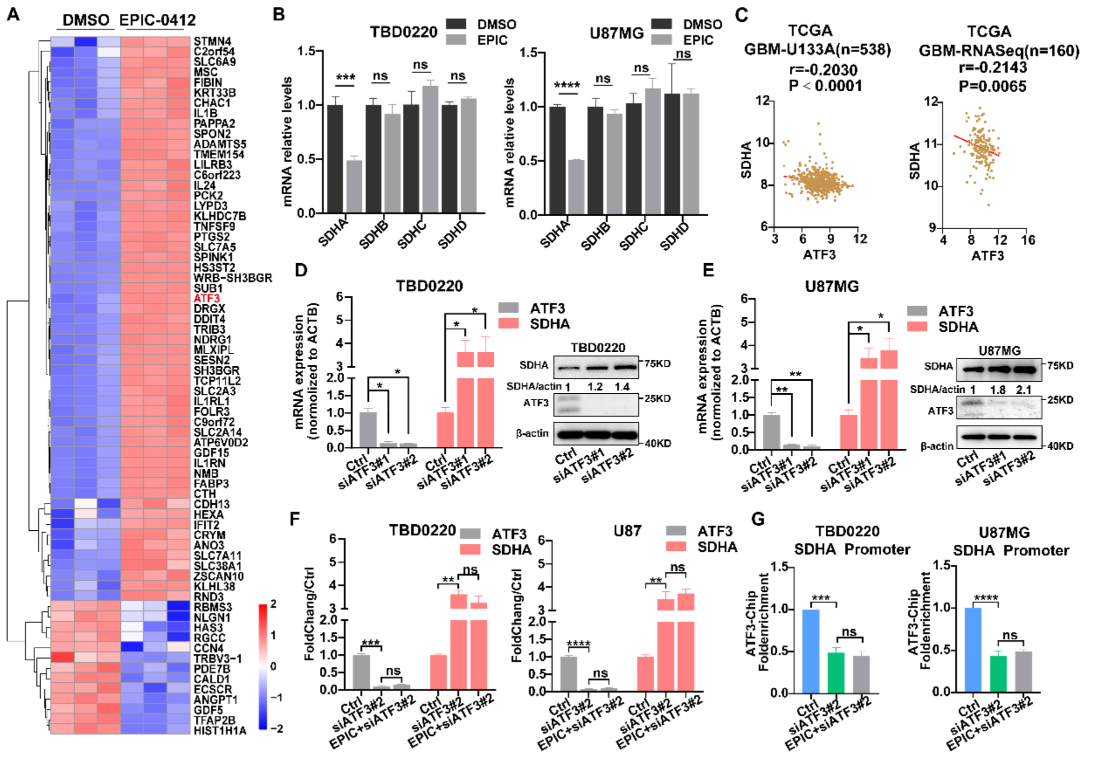

Figure S2. **(A)** Heatmap of differentially expressed genes ($\log_2 FC > 2$, $\log_2 FC < 2$, $P < 0.05$) in groups described above in ceRNA experiment ($n = 3$). **(B)** PCR analysis of SDHA, SDHB, SDHC, SDHD in TBD0220 and U87MG cells ($n = 3$). **(C)** The correlation between ATF3 and SDHA levels in the TCGA GBM database by Pearson's correlation test. **(D-E)** Verification of specificity of ATF3 siRNAs using qRT-PCR and western blot in TBD0220 **(D)** and U87MG **(E)** cells transfected with control siRNA (siCtrl) or siRNA against ATF3 for 48 h ($n = 3$). **(F)** qRT-PCR analysis of ATF3 and SDHA mRNA level after transfecting with siATF3#2 with or without treatment of EPIC (20 µm) for 48 h ($n = 3$). (G) ChIP at the SDHA promotor region in TBD0220and U87MG cells using an anti-ATF3 antibody ($n = 3$). All data are shown as the mean values ± SD, *p* values are based on two-way ANOVA. ****$p < 0.0001$, ***$p < 0.001$, **$p < 0.01$, *$p < 0.05$; ns, nonsignificant.

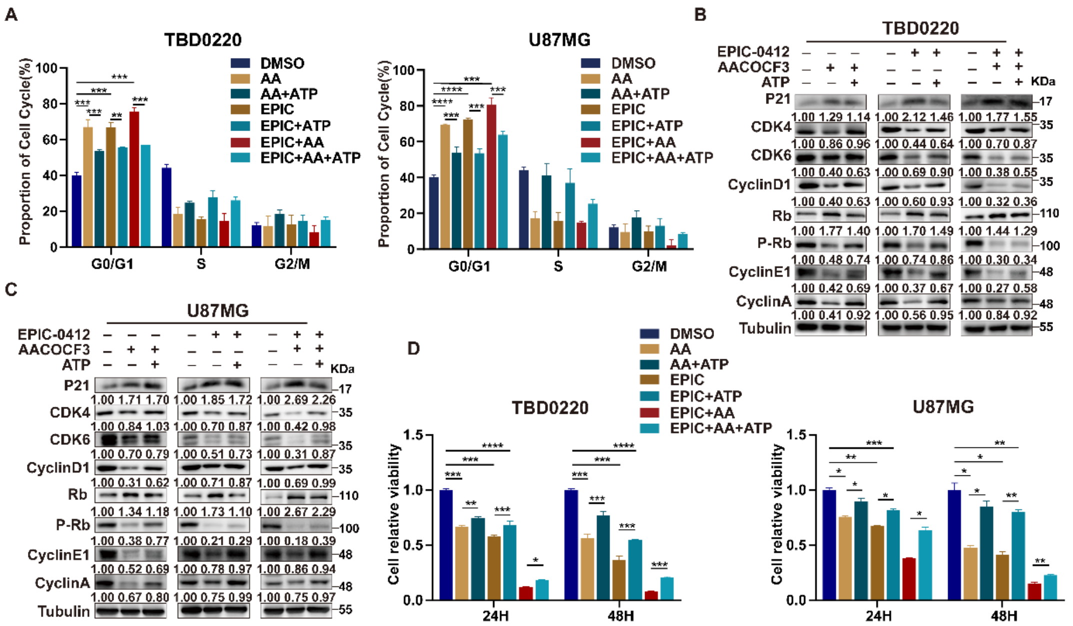

Figure S3. (A) TBD0220 and U87MG cells with different treatments and the addition of 50μM ATP for 48 h. Cell cycle distribution was monitored by flow cytometry by using propidium iodide staining. Representative histogram data are shown (n = 3). **(B) (C)** Representative western blotting showing the expression of p21 and Rb and their downstream targets in TBD0220 and U87MG cells treated or not treated with AA (25 μM), EPIC (20 μM) or EPIC + AA and ATP (50 μM) for 48 h. The results were normalized to Tubulin with the control group as 1. Protein expression was quantified by ImageJ. **(D)** Cell relative viability was measured by CCK8 assay in indicated time. Representative histogram data are shown (n = 3). All data are shown as the mean values ± SD, *p* values are based on two-way ANOVA. *****p*< 0.0001, ****p* < 0.001, ***p* < 0.01, **p* < 0.05.

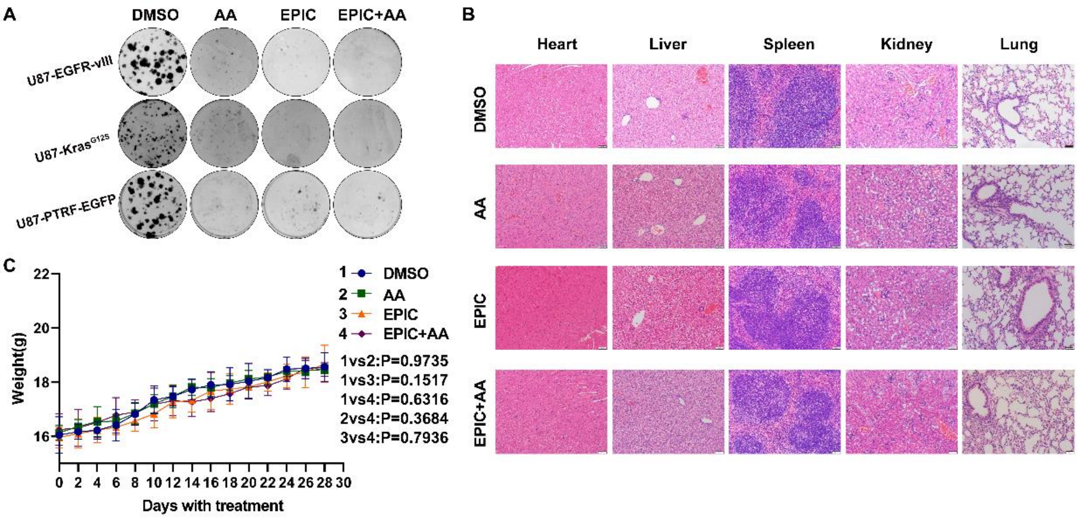

Figure S4. (A) The colony formation of cells treated with DMSO, AA, EPIC and the combination treatment of AA (25 μM) or/and EPIC (20 μM) for 14 days. **(B)** Images of HE staining of the main organs of mice after 28 days of different treatment. Scale bar: 50 μm. **(C)** Graphic representation of body weight of animals over time from all groups (n = 6).

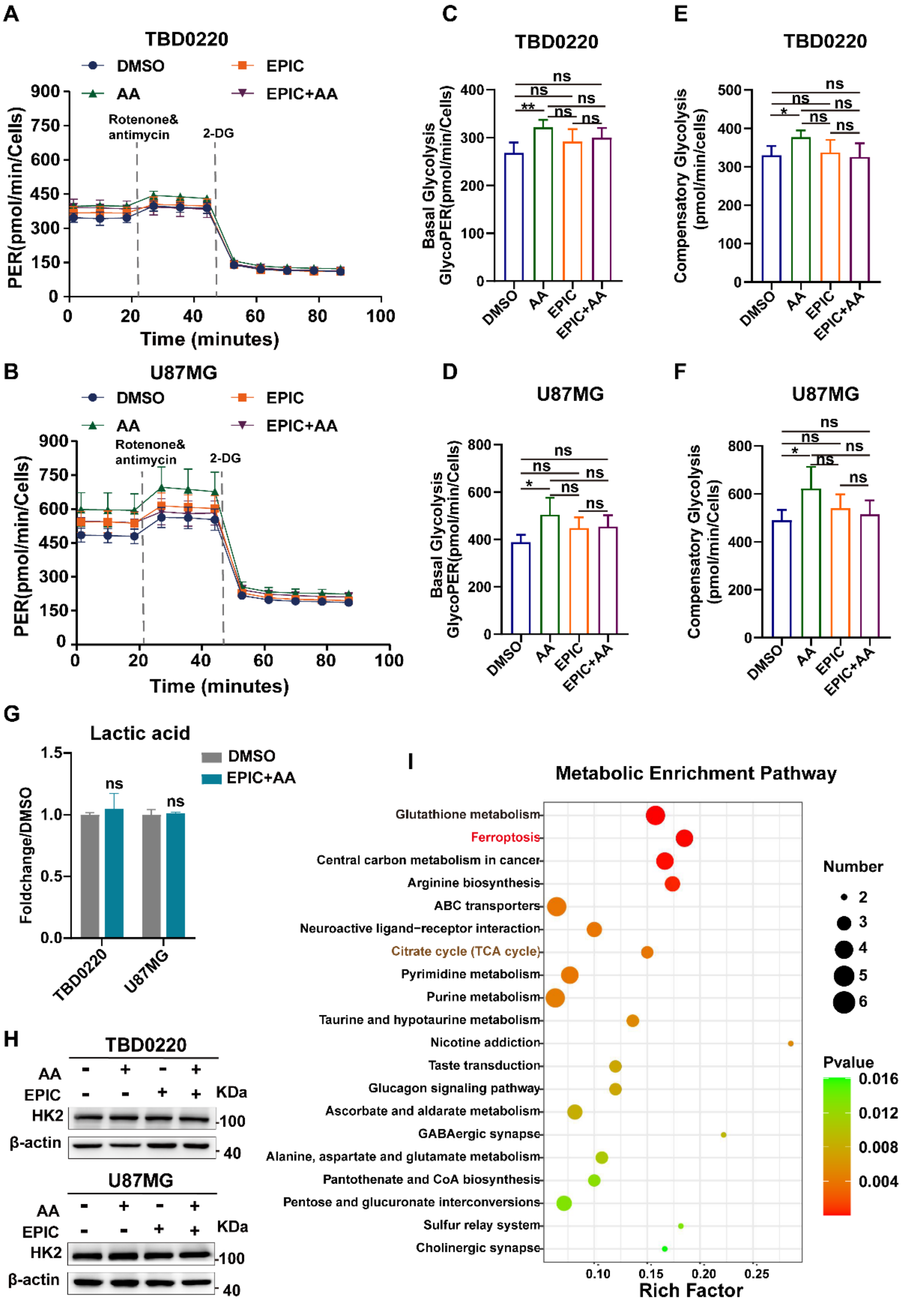

Figure S5. The combination therapy of EPIC and AA exerts no effect on glycolysis. (A-B) TBD0220 **(A)** and U87MG **(B)** cells are analyzed by glycolysis rate test on a Seahorse Metabolic Analyzer, and the results were

plotted. The addition of metabolic inhibitors is indicated (n = 3-4). **(C-D)** All groups basal glycolysis rates in TBD0220 cells **(C)** and in U87MG cells **(D)** (n = 3-4). **(E-F)** All groups Compensatory glycolysis rate in TBD0220 cells **(E)** and in U87MG cells **(F)** (n = 3-4). **(G)** Lactate was quantified by NRM metabolites detection in TBD0220 and U87MG cell lines treated with DMSO or EPIC (20 μM) + AA (25 μM) for 48 h (n = 3). **(H)** Western blotting analysis of HK2 expression in TBD0220 and U87MG cell. **(I)** Pathway analysis of significantly affected metabolites in TBD0220 cells treated with AA via non-targeted metabolomic analysis (FC > 2, $p < 0.05$ by Student's t-test). All data are shown as the mean values ± SD, *p* values are based on one-way or two-way ANOVA. $^{**}p < 0.01$, $^{*}p < 0.05$; ns, nonsignificant.

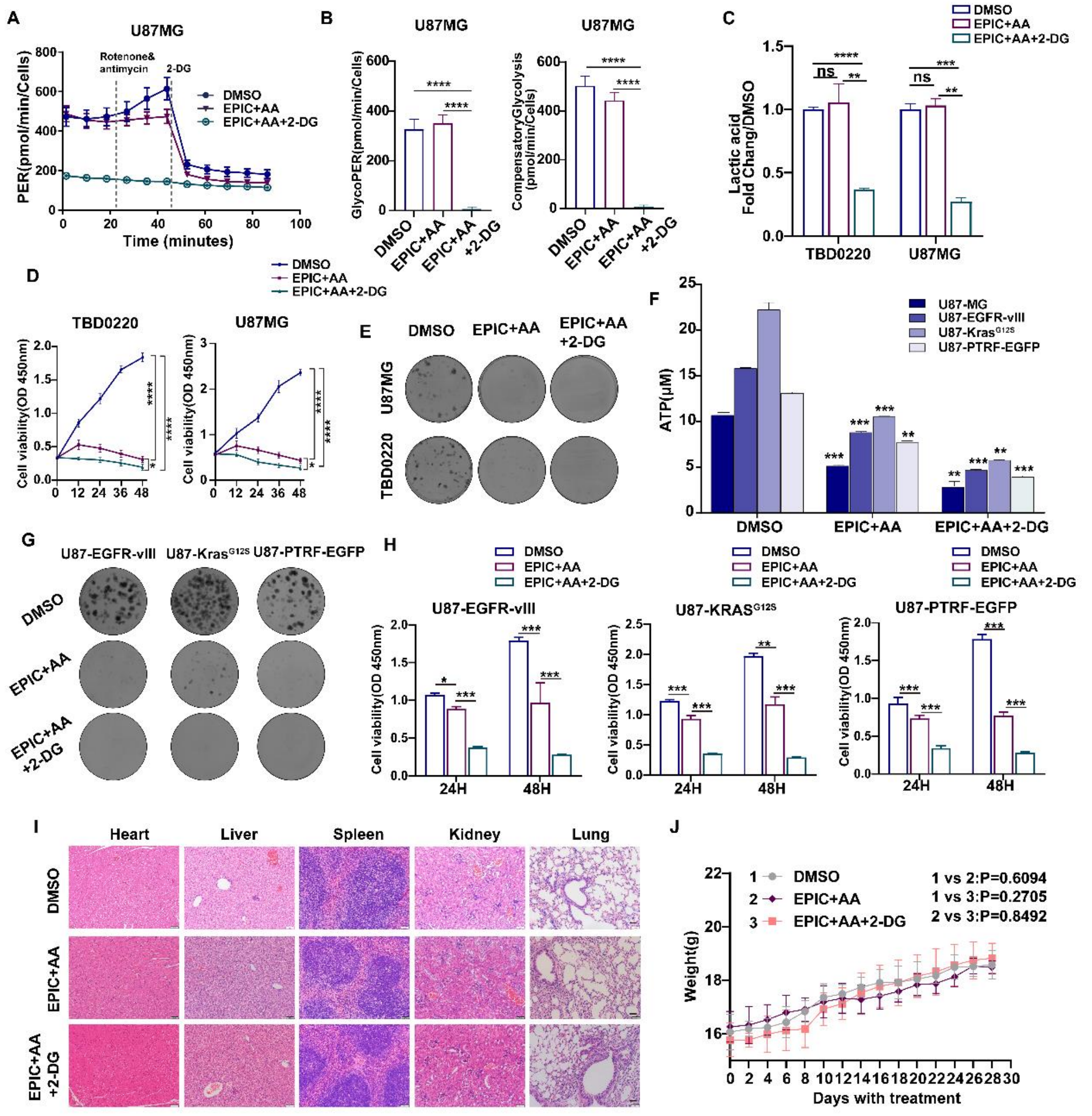

Figure. S6 (A) Proton efflux rate (PER) was measured by glycolysis rate kit by Seahorse Analyzer in U87MG cells, and the results were plotted. The addition of metabolic inhibitors is indicated (n = 3-4). **(B)** Measurements of glycoPER and compensatory glycolysis rate in U87MG cells (n = 3-4). **(C)** Lactate was quantified by NRM metabolites detection in TBD0220 and U87MG cell lines treated with DMSO or EPIC (20 μM) + AA (25 μM), EPIC (20 μM) + AA (25 μM) + 2-DG (50 mM) for 48 h (n = 3). **(D)** The cell viability of TBD0220 and U87MG cells with dual treatment or trigeminy

therapy at indicated time (n = 3). **(E)** Colony formation assays were performed with dual therapy or trigeminy therapy in TBD0220 and U87MG cells for 14 days. **(F)** ATP concentration measurements of U87MG cells (cells transduced with empty vector or corresponding lentivirus) under dual-therapy or triple therapy (n = 3). **(G-H)** Colony formation assays **(G)** and CCK-8 assay **(H)** of U87MG cells transduced with corresponding lentivirus after treatment. **(I)** Images of HE staining of the main organs of mice after 28 days of different treatment. **(J)** Graphic representation of body weight of animals over time from all groups (n = 6). All data are shown as the mean values ± SD, *p* values are based on one-way or two-way ANOVA. ****$p < 0.0001$, ***$p < 0.001$, **$p < 0.01$, *$p < 0.05$; ns, nonsignificant. Scale bar: 50 μm.
